# Syntheses, Geometric and Electronic Structures of Inorganic Cumulenes

**DOI:** 10.1021/jacs.4c13231

**Published:** 2024-11-04

**Authors:** Jianqin Tang, Chenyang Hu, Agamemnon E. Crumpton, Maximilian Dietz, Debotra Sarkar, Liam P. Griffin, Jose M. Goicoechea, Simon Aldridge

**Affiliations:** † Inorganic Chemistry Laboratory, Department of Chemistry, University of Oxford, South Parks Road, Oxford, OX1 3QR, United Kingdom; ‡ Department of Chemistry, Indiana University, 800 E. Kirkwood Ave, Bloomington, Indiana 47405, United States

## Abstract

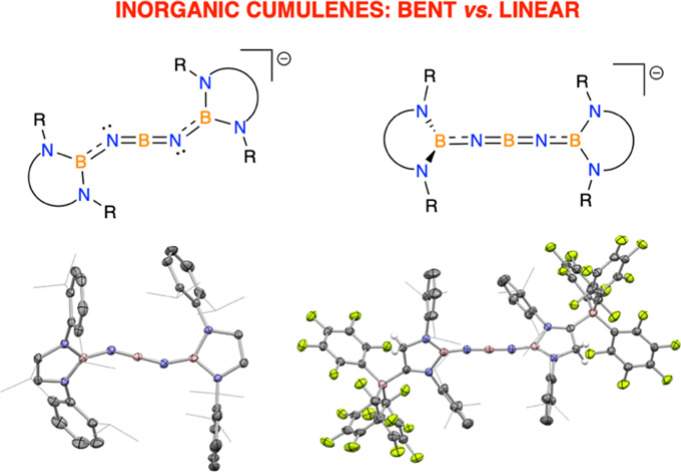

Molecular chains of two-coordinate carbon atoms (cumulenes) have long been targeted, due to interest in the electronic structure and applications of extended π-systems, and their relationship to the carbon allotrope, carbyne. While formal (isoelectronic) B=N for C=C substitution has been employed in two-dimensional (2-D) materials, unsaturated one-dimensional all-inorganic “molecular wires” are unknown. Here, we report high-yielding synthetic approaches to heterocumulenes containing a five-atom BNBNB chain, the geometric structure of which can be modified by choice of end group. The diamido-capped system is bent at the 2-/4-positions, and natural resonance theory calculations reveal significant contributions from B=N(:)–B≡N–B resonance forms featuring a lone pair at N (consistent with observed N-centered nucleophilicity). Molecular modification to generate a linear system best described by a B=N=B=N=B resonance structure involves chemical transformation of the capping groups (using B(C_5_F_5_)_3_) to enhance their π-acidity and conjugate the N-lone pairs.

Cumulenes are a family of carbon-rich molecules featuring a contiguous chain of two-coordinate carbon atoms terminated at each end by a three-coordinate carbon center. A chain of *n* + 1 carbon atoms linked via *n* C=C double bonds in this way defines an [*n*]cumulene (*n* ≥ 3), with examples featuring “simple” aryl end groups typically adopting geometrically linear structures (e.g., **I**, Figure 1).^1−3^ The rigid, conjugated frameworks of these systems have attracted interest in the context of applications as semiconductors,^4,5^ and as single-molecule wires,^6−8^ as well as models for the carbon allotrope carbyne.^9,10^

**Figure 1:**
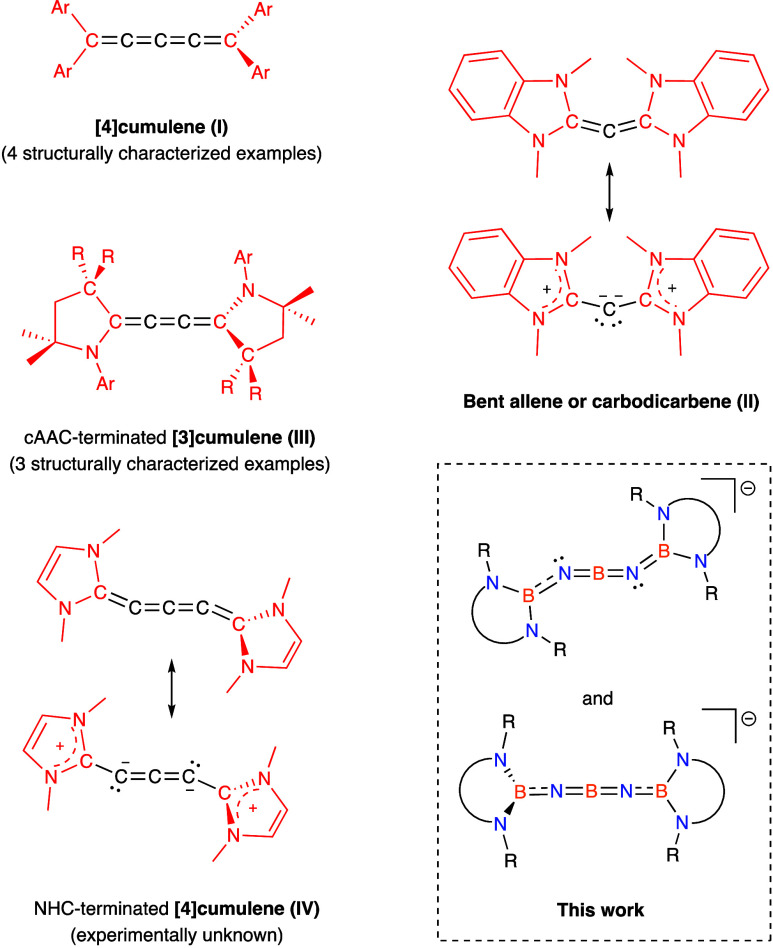
Cumulene and related systems of relevance to the current study.

More recently, [*n*]cumulenes featuring CX_2_ end groups formally derived from *cyclic* carbenes have attracted significant interest due to their ability to stabilize alternative nonlinear geometries within the *n* – 1 two-coordinate carbons of the “encapsulated” fragment. Although not strictly cumulenes (since *n* < 3), this scenario is exemplified by related bent allene systems (e.g., **II**),^11,12^ which can also be described in terms of a contributing carbo-dicarbene resonance structure. Recent quantum chemical calculations suggest that the π-acceptor capabilities of the carbene fragment are crucial in determining the stability of a linear geometry relative to alternative bent structures featuring lone pair character at the 2-position. As such, the relative π-acceptor capabilities CAr_2_ > cAAC > NHC (cAAC = cyclic amino alkyl carbene; NHC = *N*-heterocyclic carbene) suggests that nonlinear structures are most likely to be encountered with NHC termini. Consistently, each of the (three) cAAC-terminated [3]cumulenes (**III**) reported to date is linear,^13−15^ in similar fashion to systems featuring CAr_2_ end units, while NHC-terminated [4]cumulenes (**IV**) are calculated to possess bent ground-state geometries.^16^ That said, no examples of NHC terminated cumulenes of *any* chain length (i.e., *n* ≥ 3) have yet been structurally characterized to validate these claims.

The isolobal substitution of C=C for B=N dinuclear units represents a powerful design formalism that has been widely exploited in the synthesis of conjugated 2D materials derived from polycyclic aromatic hydrocarbons.^17−19^ The application of this approach to cumulenic systems, however, has not been developed, despite the possibility for novel electronic properties stemming from the conjugation of *polar* unsaturated B=N units. Three-atom NBN^20−22^ and BNB^23−25^ chains have been reported, but the only longer example featuring alternating BN units, is a four-atom chain stabilized within the coordination sphere of a transition metal.^26^ We have recently been interested in the synthesis of unsaturated compounds integrating BN units within an extended (conjugated) chain, and the modulation of electronic properties that result.^27^ As part of these studies, we report here the first example of an inorganic analogue of a cumulene, a system with features a five-atom BNBNB chain and which, depending on the nature of the end groups, can possess either a bent or linear geometric structure.

Double deprotonation of boryl-substituted diamino(bromo)borane **1** (see Scheme S1) using 2 equiv of K[N(SiMe_3_)_2_] generates K[{(HCDippN)_2_}BNBNB{(NDippCH)_2_}] (**2**) in 66% yield (Scheme 1). **2** has been characterized by multinuclear NMR spectroscopy, elemental microanalysis, and single-crystal X-ray diffraction (XRD) (Figure 2a). The simple pattern of ^1^H NMR signals (e.g., one CH and two CH_3_ signals for the Dipp substituents) implies rapid exchange of the K^+^ counterion between equivalent sites on the NMR timescale. The ^11^B NMR signal for the diazaborolyl capping moieties, observed at δ_B_ = 21.1 ppm, is found at a similar chemical shift to that for **1** (δ_B_ = 22.2 ppm), but the resonance associated with the central (two-coordinate) boron center is too broad to be resolved from the baseline.

**Scheme 1:**
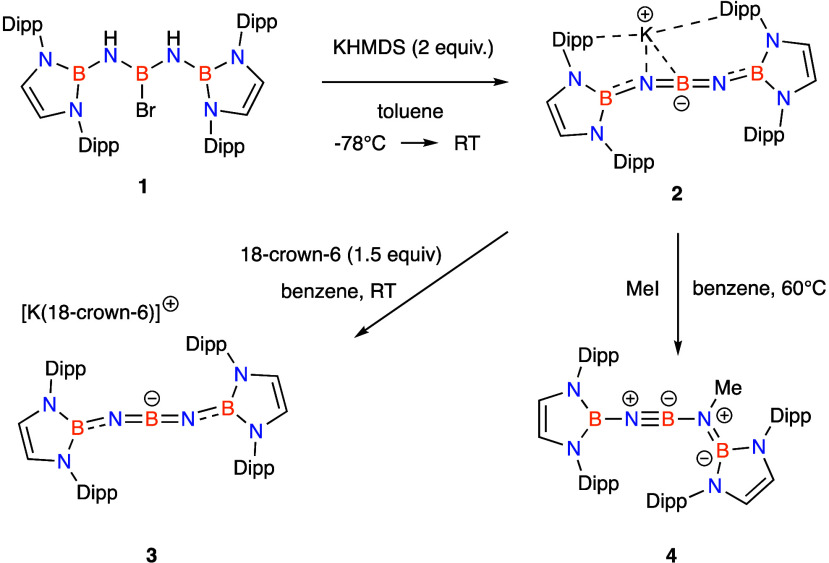
Syntheses of Compounds **2**–**4** from Boryl-Functionalized Diamino(bromo)borane, **1**

**Figure 2:**
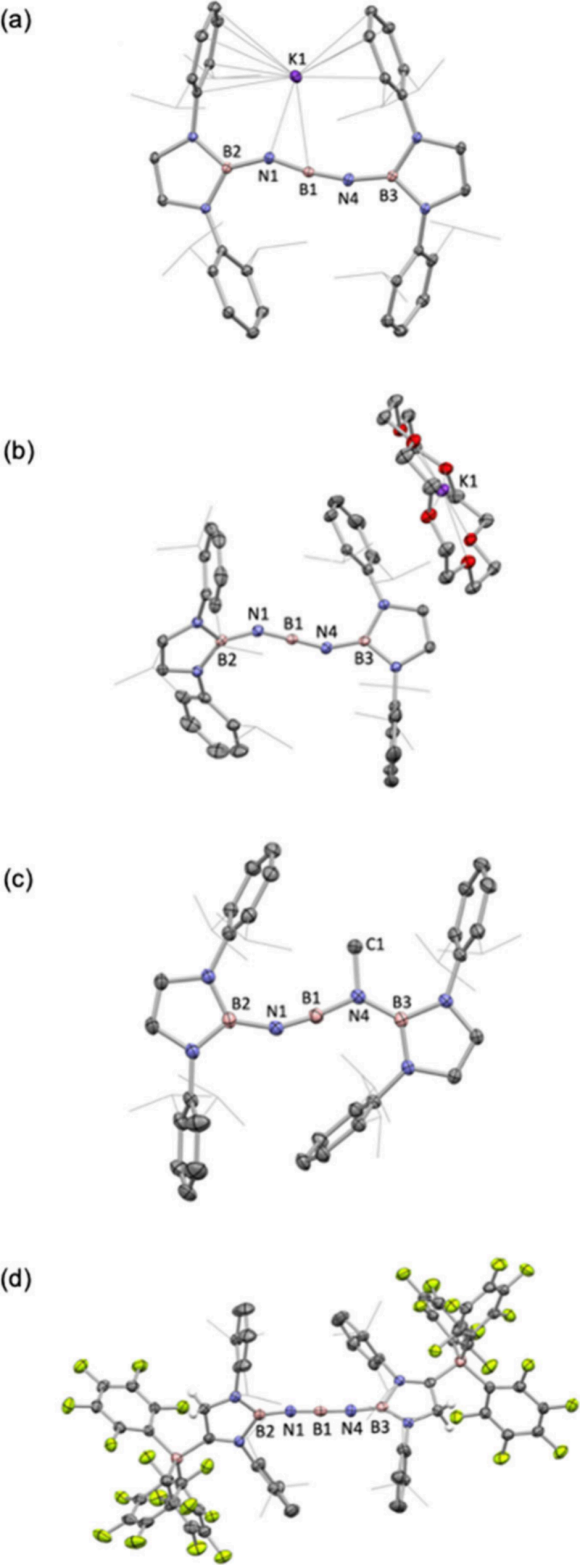
Molecular structures of (a) **2**, (b) **3**, (c) **4**, and (d) the anionic component of **5** in the solid state, as determined by X-ray crystallography. Key bond lengths and angles are listed in Table 1.

Sequestration of K^+^ from **2** can readily be effected by the use of 18-crown-6 (1,4,7,10,13,16-hexaoxa-cyclooctadecane) to yield [K(18-crown-6)][{(HCDippN)_2_}BNBNB{(NDippCH)_2_}] (**3**), which can be obtained as single crystals from benzene solution, and shown to consist (in the solid state) of well-separated anionic and cationic components (Scheme 1 and Figure 2b). **3** has been characterized in similar fashion to **2**, and gives rise to analogous patterns of signals in the respective ^1^H and ^13^C NMR spectra; the ^11^B signal corresponding to the central boron atom is again too broad to be resolved.

Preliminary probes of the nucleophilic reactivity of the anionic component of **2**/**3** were carried out by exposure to MeI. These reactions proceed via nucleophilic attack by one of the exocyclic nitrogen atoms, leading to the formation of {(HCDippN)_2_}BNBN(Me)B{(NDippCH)_2_} (i.e., (boryl)NBN(Me)(boryl), **4**). **4** has been characterized in the solid state by X-ray crystallography (Figure 2c), and in solution by multinuclear NMR methods, and can plausibly described as an iminoborane (XB≡X′) featuring amido and boryl X/X′ substitutents, respectively.

The structures of compounds **2**–**4** have been determined crystallographically. Each features a five-atom BNBNB framework capped at either end by two formally dianionic diazabutadiene moieties. The structure of **3**, in particular, allows for discussion of geometric parameters of the BN-containing anion free from the perturbation of the associated countercation. The geometry around the central boron atom (B(1)) is linear (179.7(3)°), while the B–N–B angles around the exocyclic nitrogens (N(1) and N(4)) are narrower (151.3(2)° and 152.0(2)°; Table 1). This bent geometry is consistent with that calculated for the (hypothetical) NHC-terminated [4]cumulene {(HCMeN)_2_C}=C=C=C={C(NMeCH)_2_} which features an angle at C(2) of 136.7°(albeit with a relatively flat potential energy profile for bending in the region of 120°–180°).^16^

**Table 1:** Selected Bond Lengths and Bond Angles for the Central BNBNB Unit of Compounds **2**–**5**

parameter	**2**	**3**	**4**	**5**
B(1)–N(1) bond (Å)	1.319(2)	1.308(4)	1.265(3)	1.294(7)
B(1)–N(4) bond (Å)	1.286(2)	1.307(4)	1.398(3)	1.279(7)
N(1)–B(1)–N(4) bond angle (deg)	171.1(1)	179.7(3)	172.1(3)	177.9(4)
B(2)–N(1) bond (Å)	1.393(2)	1.379(3)	1.409(3)	1.332(7)
B(3)–N(4) bond (Å)	1.381(1)	1.383(4)	1.446(3)	1.340(7)
B(2)–N(1)–B(1) bond angle (deg)	147.0(1)	152.0(2)	153.8(2)	172.8(4)
B(1)–N(4)–B(3) bond angle (deg)	164.2(1)	151.3(2)	126.9(2)	173.8(4)

The B–N distances associated with B(1) are short (1.307(4), 1.308(4) Å), with those involving B(2)/B(3) being somewhat longer (1.379(3), 1.383(4) Å), consistent with the increased coordination number at boron. In a broader context, the B(1)–N distances can be compared to compounds containing formal B≡N triple bonds, which are markedly shorter (e.g., 1.228(3) Å for Mes*BNNBMes*, where Mes* = 2,4,6-^*t*^Bu_3_C_6_H_2_),^28−32^ and to the BN distance between the two-coordinate boron and nitrogen centers in methylated derivative **4** (1.265(3) Å), which can also be described in terms of a resonance structure incorporating a B≡N triple bond. However, the B(1)–N distances are noticeably shorter than typical B=N double bonds, e.g., those found in the linear BN_2_^3–^ trianion (1.358(6) Å),^33,34^ or in aminoboranes (e.g., 1.376(4) Å for ^*i*^Pr_2_NB(C_6_F_5_)_2_).^35^ Consistently, the solid-state IR spectrum of **3** shows a band at 1890 cm^–1^ (Figure S5), which is assigned to the antisymmetric BN stretching vibration of the central NBN unit, based on quantum chemical simulation. This can be compared to the antisymmetric BN stretch measured for BN_2_^3–^ (1664 cm^–1^),^36^ and bands associated with the stretching of B≡N bonds, which fall between 1879 cm^–1^ and 1982 cm^–1^.^37−39^

To better understand the electronic structure of **3**, density functional theory (DFT) calculations coupled with natural bond orbital (NBO) and natural resonance theory (NRT) analyses were carried out (Figure 3). The Mayer bond indices (MBIs) calculated for the two central bonds (B(1)–N(1) and B(1)–N(4)) are 1.77, while the corresponding values for B(2)–N(1) and B(3)–N(4) are lower (1.38), although still greater than those within the diazaborolyl heterocycles (1.06). NPA charge analysis determines (as expected) that the N atoms bear the negative charge (−1.29 au for N1/N4), while the central boron atom bears a positive charge (+1.12 au) and the three-coordinate borons charges of +1.04 au (Figure 3a). As such, the N-centered nucleophilic reactivity of these systems (i.e., reactions with electrophiles such as MeI) is readily understood.

**Figure 3:**
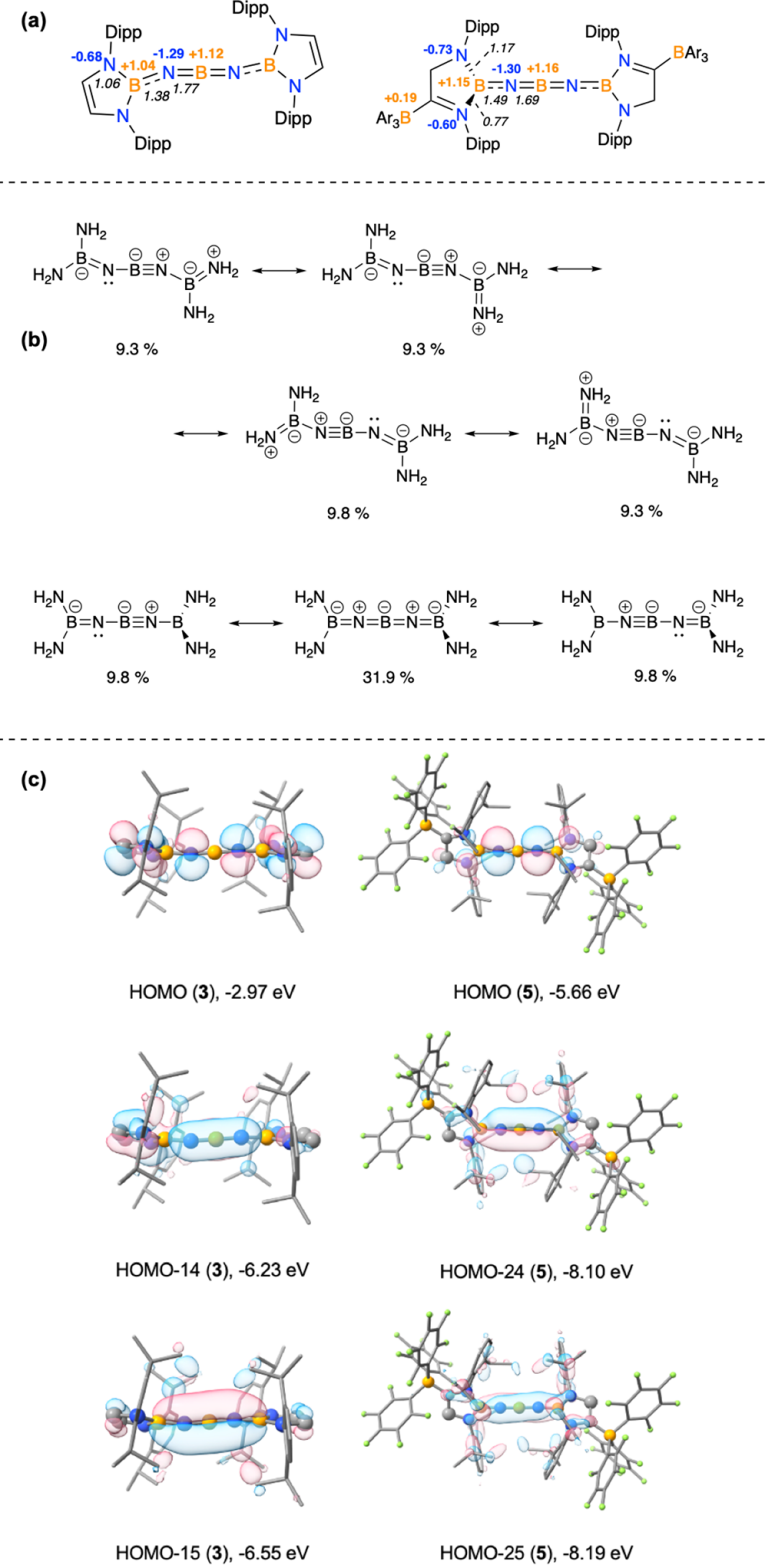
(a) Mayer bond indices (italics) and selected NPA charges for the anionic components of **3** and **5**; (b) key resonance structures for a simplified model of the anionic BNBNB chain determined using NRT for both bent and linear isomers; (c) key molecular orbitals for the anionic components of **3** and **5** (iso-surface value = 0.03).

Natural resonance theory (NRT) calculations were carried out to assess the relative contributions of different valence bond forms to the overall electronic structure of **3** (Figure 3b). We exploited the simplified diminoborane model [(H_2_N)_2_BNBNB(NH_2_)_2_]^−^, in order to avoid complications stemming from the large number of resonance structures associated with pendant aromatic rings, and focus instead on the key BNBNB unit. We examined resonance structures for both bent and linear geometries: for the linear geometry the dominant contributing structure (amounting to 31.9%) features a contiguous chain of double bonds, B=N=B=N=B, while the bent geometry is described best by four (essentially equivalent) B=N(:)–B≡N–B resonance forms (summing to 37.7%), which involve a B≡N triple bond and a lone pair at the other N center.^16^ Taken together, these four resonance forms imply that there is BN π bonding across the central NBN unit in two orthogonal planes, while the BN units at the end of the chain are defined by π bonding in only one plane.

The molecular orbital picture of **3** reveals delocalized π systems along the central BNBNB fragment. The HOMO is, in effect, the out-of-phase combination of the nitrogen-based lone pairs (*E* = −2.97 eV, Figure 3c), while much lower-lying orbitals possess highly delocalized BN bonding character. As such, the HOMO–15 (*E* = −6.55 eV) is the in-phase combination of *p*π orbitals spanning the entire five-atom BNBNB chain, while the HOMO–14 (*E* = −6.23 eV) lies approximately orthogonal to it, and spans the central NBN core, lying closer to coplanar with the capping diazaborolyl heterocycles.

Attempts to probe the electrochemical oxidation of **2** or **3** reveal only features consistent with irreversible processes, and no clean reactivity is observed with a range of one- and two-electron *chemical* oxidants. On the other hand, the reaction of **2** with B(C_6_F_5_)_3_ proceeds cleanly to give a more unsymmetrical product, as implied by ^1^H NMR measurements; single crystals could be obtained from pentane solution and the structure of the ion pair **5** was confirmed by X-ray crystallography (Scheme 2 and Figure 2d).

**Scheme 2:**
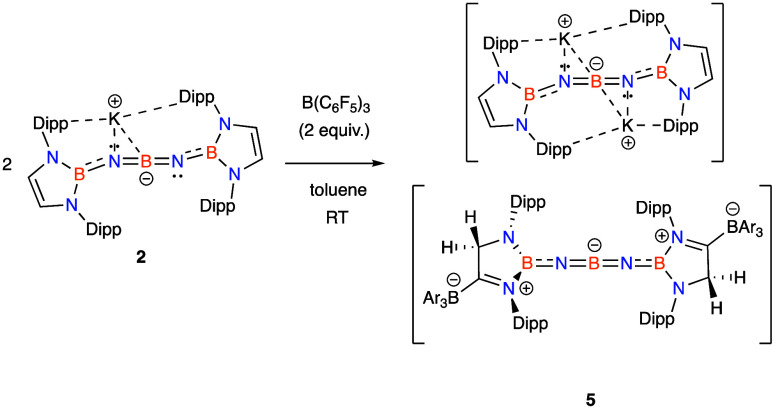
Reaction of **2** with BAr_3_ (Ar = C_6_F_5_) To Generate a Linear BNBNB Chain

While the cationic component is straightforwardly derived from **2** by uptake of the K^+^ counterion from a second equivalent of the starting material, the anionic component has been transformed by the assimilation of a molecule of the borane at one of the backbone positions of each boryl heterocycle. Attack by the borane at the backbone carbon rather than at nitrogen is presumably sterically driven (cf. the reaction with MeI), and finds precedent in boryl and carbene ligand chemistry.^40,41^ This takeup of B(C_6_F_5_)_3_, together with an accompanying 1,2-hydrogen shift leads to significant modification in the capping units at each end of the BNBNB chain. Most importantly, it leads to loss of the C=C bond within the heterocycle, with accompanying formation of a C=N bond involving one of the heterocyclic nitrogen atoms (*d*(C(3)N(2)) = 1.301(5) Å). This double bond effectively arises through sharing of the N-lone pair at N(2) with C(3), which, in turn, would be expected to render the boron atoms within the heterocycles (B2/3) significantly less π-stabilized.^40,41^ As such, the capping heterocycles in the anionic component of **5** are significantly more π acidic than those in **2**/**3**, in a manner reminiscent of the difference between cAAC and NHC carbenes (single N-donor π-stabilized vs doubly stabilized).^42^ The most obvious structural consequence of this change is that the BNBNB moiety becomes linear, with the crystallographically determined angles at the nitrogen atoms being 173.8(4)° and 172.8(4)° (cf. ca. 150° for **3**); the central boron atom remains linear (177.9(4)°, cf. 179.7(3)° for **3**).

With reference to quantum chemical calculations carried out on the isoelectronic all-carbon [4]cumulenes,^16^ this geometric modification can be understood by consideration of the enhanced π-acceptor capabilities of the capping groups, which effectively lead to the lone pairs found at nitrogen in compounds **2**/**3** being conjugated into the BNBNB chain (Figure S6). This change is reflected in shorter chain-end BN distances for the anionic component of **5** (1.332(7)/1.340(7) Å vs 1.379(3)/1.383(4) Å for **3**) and slightly higher MBIs (1.49 vs 1.38). The molecular orbital picture for the anionic component of **5** is also consistent with the existence of a linear BNBNB chain featuring an approximately orthogonal alignment of the capping heterocycles (torsion angle = 72.7°). As such, the near degenerate HOMO–24 and HOMO–25 orbitals (found at −8.10 and 8.19 eV, respectively) define orthogonal π-bonding orbitals spanning the two (overlapping) four-atom BNBN fragments (Figure 3c).

## Supporting Information Available

Synthetic and characterizing data for novel compounds; representative spectra; details of quantum chemical calculations and X-ray crystallography (PDF)

## ABBREVIATIONS

cAAC: cyclic amino alkyl carbene
NHC: *N*-heterocyclic carbene
Dipp: 2,6-diisopropylphenyl
