# Supplementary material for: Syntheses, Geometric and Electronic Structures of Inorganic Cumulenes
Source: J Am Chem Soc. 2024 Nov 4;146(45):30778–83. doi: 10.1021/jacs.4c13231 (PMC11565641; doi:10.1021/jacs.4c13231)
Supplement: Supplementary file 1 — ja4c13231_si_001.pdf [file ja4c13231_si_001.pdf]

Syntheses, geometric and electronic structures of inorganic cumulenes

Jianqin Tang, Chenyang Hu, Agamemnon E. Crumpton, Maximilian Dietz, Debotra Sarkar, Liam P. Griffin, Jose M. Goicoechea, Simon Aldridge

Inorganic Chemistry Laboratory, Department of Chemistry, University of Oxford, South Parks Road, Oxford, OX1 3QR (UK)

Department of Chemistry, Indiana University, 800 E. Kirkwood Ave, Bloomington, IN 47405 (USA)

Supporting information (27 pages)

1. General considerations 2

2. Preparation of starting materials 2

3. Synthetic procedures, characterising data and representative spectra of new compounds 3

4. Details of X-ray crystallography 15

5. Details of quantum chemical calculations 19

6. References for supporting information 26

1. General considerations

All experiments were carried out under an atmosphere of dry argon or dinitrogen using standard Schlenk line or dry-box techniques. Solvents were degassed by sparging with argon and dried by passing through a column of appropriate drying agent using a commercially available Braun SPS and stored over potassium mirror under argon in a Teflon valve ampoule. NMR spectra were measured in d_6-benzene, with the solvent being dried over calcium hydride or molten potassium respectively, distilled, degassed by three freeze-pump-thaw-cycles and stored over 3 Å sieves. NMR samples were prepared under argon in 5 mm Wilmad 507-PP tubes fitted with J. Young Teflon valves. ^{1}H and $^{13}C\{^{1}H\}$ NMR spectra were measured on a Bruker Avance III HD Nanobay 400 MHz or Bruker Avance III 500 MHz spectrometer, and referenced internally to residual protio-solvent (^{1}H) or solvent (^{13}C) resonances; resonances are reported relative to tetramethylsilane (δ = 0 ppm). Assignments were confirmed using two-dimensional ^{1}H-^{1}H, ^{13}C-^{1}H, NMR correlation experiments. Chemical shifts are quoted in δ (ppm) and coupling constants in Hz. The reported yields are the yields obtained after crystallisation and subsequent measurement of the material by single crystal-ray diffraction if not stated otherwise. Elemental analyses were carried out by London Metropolitan University or Elemental Microanalysis Ltd, Okehampton, Devon, UK.

2. Preparation of starting materials

$(HCDippN)_2BNH_2$ was prepared by the literature procedure.[1]

3. Synthetic procedures, characterising data and representative spectra of new compounds

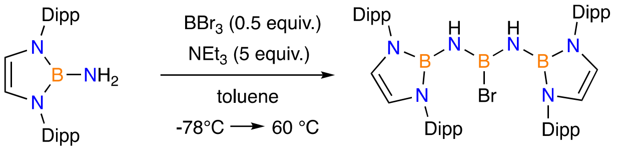

1

Scheme S1. Synthesis of $\{(HCDippN)_2BNH\}_2BBr$, **1**.

$\{(HCDippN)_2BNH\}_2BBr$, 1. $(HCDippN)_2BNH_2$ (515.7 mg, 1.28 mmol) and NEt_3 (0.26 mL, 1.86 mmol) were dissolved in toluene (15 mL) at -78 °C and BBr_3 added dropwise. The resulting solution was stirred for 15 min at -78 °C, warmed to room temperature, and then heated at 60 °C overnight with an accompanying colour change from yellow to light brown. Volatiles were then removed in vacuo, and the residue extracted with pentane (80 mL). The resulting solution was concentrated (to ca. 15 mL) and colourless crystals of **1** were obtained upon standing at -30 °C which were suitable for X-ray crystallography. These were isolated by filtration and washed with cold pentane (2 x 5 mL).

Yield: 402.3 mg (70.3 %)

^{1}H NMR (500 MHz, C_6D_6, 298 K): δ_H 1.13 (d, $^{3}J_{H,H}$ = 7.0 Hz, 24H, CH_3 of Dipp), 1.16 (d, $^{3}J_{H,H}$ = 7.0 Hz, 24H, CH_3 of Dipp), 3.13 (br, 2H, NH), 3.17 (sept, 8H, C$\underline{H}$$(CH_3)_2$ of Dipp), 5.98 (s, 4H, CH of boryl), 7.08 (m, 8H, m-ArH of Dipp), 7.19 (m, 4H, p-ArH of Dipp).

$^{11}B\{^{1}H\}$ NMR (160 MHz, C_6D_6, 298 K): δ_B 22.2 (boryl), 28.3 (N-B-N)

$^{13}C\{^{1}H\}$ NMR (126 MHz, C_6D_6, 298 K): δ_C 23.8, 24.9 (CH_3 of Dipp), 28.6 ($\underline{C}H(CH_3)_2$ of Dipp), 117.9 (CH of boryl), 123.8 (m-Ar of Dipp), 127.7 (p-Ar of Dipp), 138.5 (CN of Dipp), 146. 6 (o-Ar of Dipp).

Elemental microanalysis: calc. for $C_{52}H_{74}B_3BrN_6$ (%): C 69.74 H 8.33 N 9.38; meas. C 69.59 H 8.35 N 8.94.

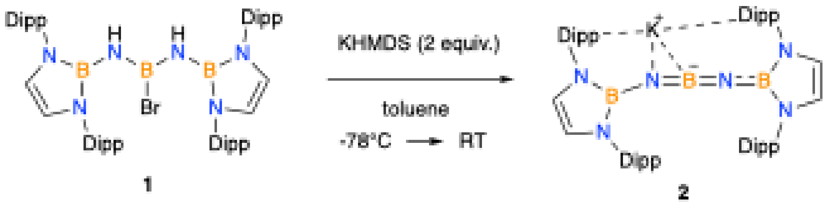

Scheme S2. Synthesis of [{(HCDippN)$_2$BN}$_2$B]K, **2**.

[{(HCDippN)$_2$BN}$_2$B]K, 2. To a solid mixture of **1** (490 mg, 0.54 mmol) and KHMDS (218 mg, 1.09 mmol) in a 100 mL Schlenk flask at -78 °C was added 40 mL toluene. The resulting solution was allowed to warm to room temperature and stirred for 12 h with formation of a brown precipitate and a colour change to light green. Volatiles were removed in vacuo and the residue extracted with pentane (120 mL). The resulting solution was concentrated to ca. 15 mL and colourless crystals of **2** were obtained which were suitable for X-ray crystallography. These were isolated by filtration and washed with cold pentane (2 x 5 mL).

Yield: 301.8 mg (65.6 %)

^{1}H NMR (500 MHz, C_6D_6, 298 K): δ_H 1.15 (d, $^{3}J_{H,H}$ = 6.8 Hz, 24H, CH_3 of Dipp), 1.21 (d, $^{3}J_{H,H}$ = 6.8 Hz, 24H, CH_3 of Dipp), 3.40 (sept, $^{3}J_{H,H}$ = 6.9 Hz, 8H,C<u>H</u>(CH_3)$_2$ of Dipp), 6.00 (s, 4H, CH of boryl), 7.02 (m, 8H, m-ArH of Dipp), 7.05 (m, 4H, p-ArH of Dipp).

^{11}B{^{1}H} NMR (160 MHz, C_6D_6, 298 K): δ_B 21.1 (boryl), not observed (N=B=N).

^{13}C{^{1}H} NMR (126 MHz, C_6D_6, 298 K): δ_C 24.0, 24.3 (CH_3 of Dipp), 28.5 (<u>C</u>H(CH_3)$_2$ of Dipp), 115.8 (CH of boryl), 123.0 (m-Ar of Dipp), 126.1 (p-Ar of Dipp), 142.2 (CN of Dipp), 148.6 (o-Ar of Dipp).

Elemental microanalysis: calc. for $C_{52}H_{72}B_3KN_6$ (%): C 73.24 H 8.51 N 9.86; meas. C 72.76 H 8.85 N 9.54.

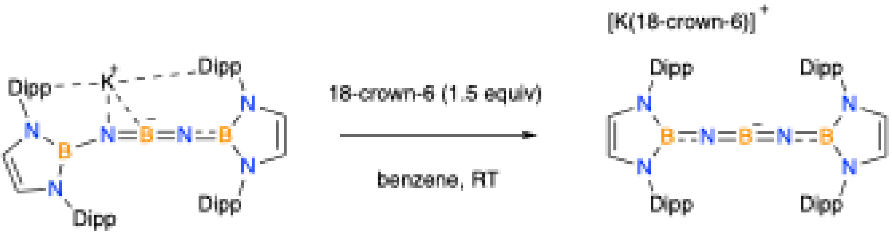

Scheme S3. Synthesis of [{(HCDippN)$_2$BN}$_2$B]K(18-crown-6), **3**.

[{(HCDippN)$_2$BN}$_2$B]K(18-crown-6), 3. 2 (15 mg, 0.017 mmol) and 18-crown-6 (4.6 mg, 0.017) were dissolved in 2 mL benzene and stirred for 2 h at room temperature. The reaction mixture was concentrated (to ca. 0.4 mL) and colourless crystals of **3** were obtained upon standing at room temperature which were suitable for X-ray crystallography. These were isolated by filtration and washed twice with cold pentane (2 x 0.1 mL) and dried under vacuum.

Yield: 17.6 mg (89.7 %)

^{1}H NMR (500 MHz, C_6D_6, 298 K): δ_H 1.36 (d, $^3J_{H,H}$ = 6.8 Hz, 24H, CH_3 of Dipp), 1.42 (d, $^3J_{H,H}$ = 6.8 Hz, 24H, CH_3 of Dipp), 3.03 (s, 18-crown-6), 3.78 (sept, $^3J_{H,H}$ = 7.0 Hz, 8H,C$\underline{H}$(CH_3)$_2$ of Dipp), 6.10 (s, 4H, CH of boryl), 7.20 (m, 4H, p-ArH of Dipp), 7.21 (m, 8H, m-ArH of Dipp).

^{11}B{^{1}H} NMR (160 MHz, C_6D_6, 298 K): δ_B 21.0 (boryl), not observed (N=B=N).

^{13}C{^{1}H} NMR (126 MHz, C_6D_6, 298 K): δ_C 24.4, 24.5 (CH_3 of Dipp), 28.7 ($\underline{C}$H(CH_3)$_2$ of Dipp), 70.2 (s, 18-crown-6), 116.4 (CH of boryl), 123.0 (m-Ar of Dipp), 125.0 (p-Ar of Dipp), 144.0 (CN of Dipp), 147.7 (o-Ar of Dipp).

Elemental microanalysis: calc. for $C_{64}H_{96}B_3KN_6O_6$ (%): C 68.82 H 8.66 N 7.52; meas. C 69.53 H 8.83 N 7.54.

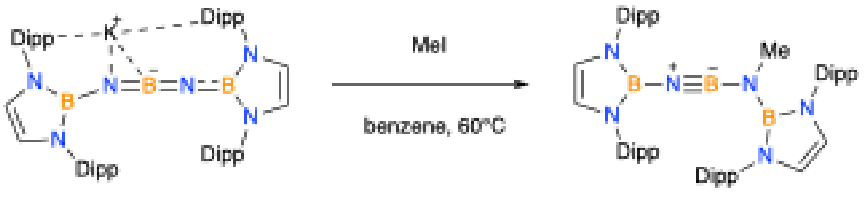

Scheme S4. Synthesis of [(HCDippN)$_2$BNBN(Me)B(NDippCH)$_2$], **4**.

[(HCDippN)$_2$BNBN(Me)B(NDippCH)$_2$], 4. 2 (20 mg 0.023 mmol) was dissolve in benzene (0.4 mL) and a drop of MeI (0.01 mL, excess) was added. The solution was heated at 60 °C overnight with the formation of a white precipitate. Volatiles were removed in vacuo and the residue extracted with pentane (4 mL). The resulting solution was concentrated to ca. 0.4 mL and colourless crystals of **4** were obtained upon standing at room temperature which were suitable for X-ray crystallography. These were isolated by filtration and washed with cold pentane (2 x 0.1 mL).

Yield: 13.5 mg (69.4 %)

^{1}H NMR (500 MHz, C_6D_6, 298 K): δ_H 1.15 (t, $^3J_{H,H}$ = 6.8 Hz, 12H, CH_3 of Dipp), 1.17 (t, $^3J_{H,H}$ = 6.8 Hz, 12H, CH_3 of Dipp), 1.23 (d, $^3J_{H,H}$ = 6.8 Hz, 12H, CH_3 of Dipp), 1.27 (d, $^3J_{H,H}$ = 6.9 Hz, 12H, CH_3 of Dipp), 1.85 (s, Me), 3.12 (sept, $^3J_{H,H}$ = 6.9 Hz, 4H,C<u>H</u>(CH_3)$_2$ of Dipp), 3.27 (sept, $^3J_{H,H}$ = 6.9 Hz, 2H,C<u>H</u>(CH_3)$_2$ of Dipp), 5.84 (s, 4H, CH of boryl), 5.96 (s, 2H, CH of boryl), 6.98 (m, 6H, ArH of Dipp), 7.11 (m, 6H, ArH of Dipp).

^{11}B{^{1}H} NMR (160 MHz, C_6D_6, 298 K): δ_B 22.0 (boryl), 19.7 (N=B).

^{13}C{^{1}H} NMR (126 MHz, C_6D_6, 298 K): δ_C 23.5, 24.4, 24.6, 24.8 (CH_3 of Dipp), 28.6, 28.8 (<u>C</u>H(CH_3)$_2$ of Dipp), 34.1 (Me), 117.2, 118.5 (CH of boryl), 123.4, 123.9, 126.9, 128.6, 137.8, 140.4, 146.1, 146.9 (Ar of Dipp).

Elemental microanalysis: calc. for $C_{53}H_{75}B_3N_6$ (%): C 76.82 H 9.12 N 10.14; meas. C 76.55 H 8.70 N 9.74.

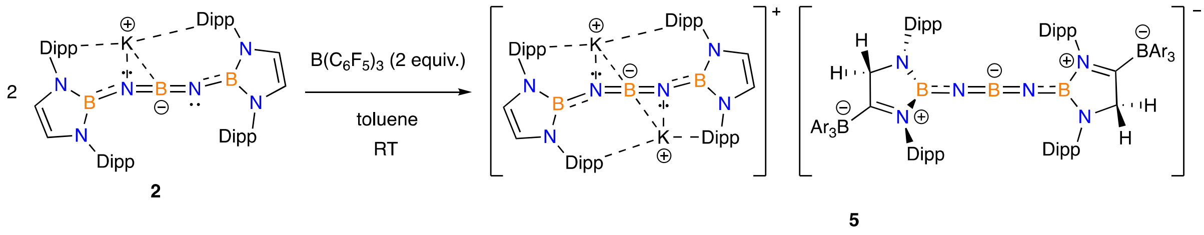

Scheme S5. Synthesis of [{$(C_6F_5)_3BCCH_2(DippN)_2BN\}_2B][\{(HCDippN)_2BN\}_2B]K_2$, **5**.

[{$(C_6F_5)_3BCCH_2(DippN)_2BN\}_2B][\{(HCDippN)_2BN\}_2B]K_2$, 5. To a solid mixture of **2** (100mg, 0.12 mmol) and $B(C_6F_5)_3$ (60 mg, 0.12 mmol) in a 25 mL Schlenk flask at room temperature was added 4 mL toluene. The solution was stirred 30 min with a colour change to yellow. Volatiles were removed in vacuo, and the residue was extracted with pentane (20 mL). The resulting solution was concentrated to ca. 5 mL and colourless crystals of **5** were obtained which were suitable for X-ray crystallography. These were isolated by filtration and washed with cold pentane (2 x 1 mL).

Yield: 76.2 mg (47.6 %)

^{1}H NMR (500 MHz, C_6D_6, 298 K): δ_H 0.94 (d and br, $^3J_{H,H}$ = 6.9 Hz, 48H, CH_3 of Dipp), 1.00 (br, 12H, CH_3 of Dipp), 1.12 (d, $^3J_{H,H}$ = 6.9 Hz, 24H, CH_3 of Dipp), 1.20 (d, $^3J_{H,H}$ = 6.8 Hz, 12H, CH_3 of Dipp), 3.08 (sept, $^3J_{H,H}$ = 6.8 Hz, 8H,C<u>H</u>$(CH_3)_2$ of Dipp), 3.16 (sept, $^3J_{H,H}$ = 6.9 Hz, 8H,C<u>H</u>$(CH_3)_2$ of Dipp), 4.53 (br, 4H, CH_2 of boryl), 5.89 (s, 4H, CH of boryl), 6.56 (br, 3H, Ar-H), 6.77 (t, 2H, Ar-H), 6.92 (m, 12H, Ar-H), 700 (m, 7H, Ar-H)

^{11}B{^{1}H} NMR (160 MHz, C_6D_6, 298 K): δ_B -15.1 ($B(C_6F_5)_3$), 20.2 (boryl), not observed (N=B=N).

^{19}F{^{1}H} NMR (470 MHz, C_6D_6, 298 K): δ_F -165.7, -163.8, -158.1, -157.6, -130.0, -126.8.

^{13}C{^{1}H} NMR (126 MHz, C_6D_6, 298 K): δ_C 22.9, 24.0, 24.1, 24.5, 25.2, 26.0 (CH_3 of Dipp), 28.6, 28.7 (<u>C</u>H$(CH_3)_2$ of Dipp), 68.1 (CH_2 of boryl), 116.3 (CH of boryl), 123.4, 124.2, 126.8, 127.7, 128.7, 128.8, 137.1, 141.3, 148.1, 148.5 (Ar of Dipp).

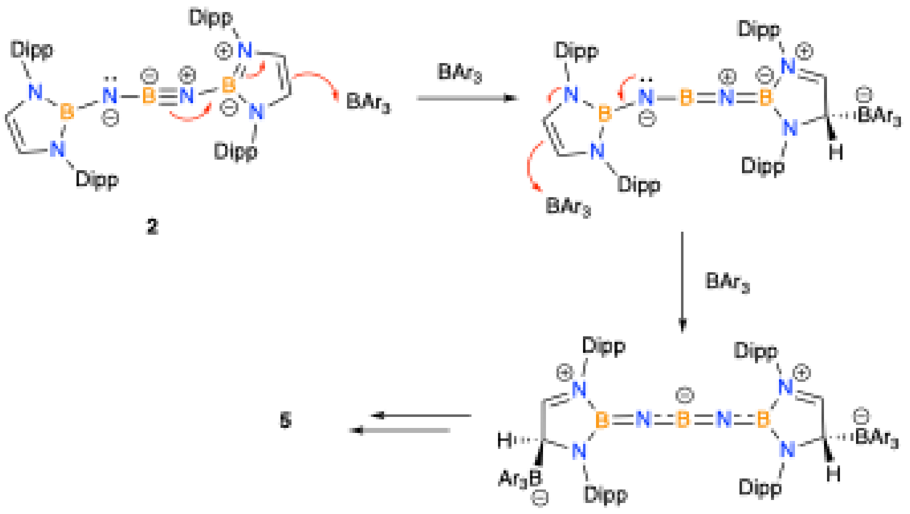

Scheme S6. Proposed (partial) mechanism for the conversion of **2** to **5** (anionic components only included for clarity).

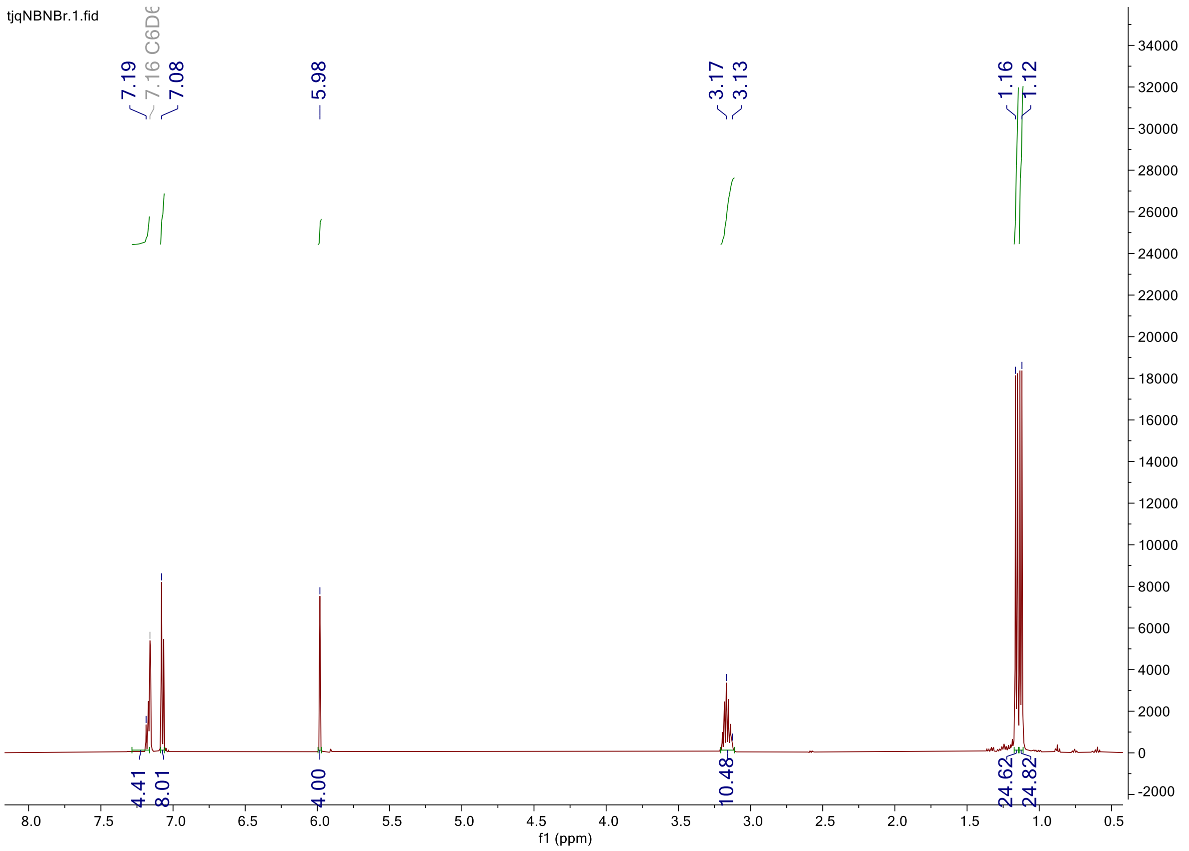

Figure S1. ^{1}H NMR spectrum of **1** (500 MHz, C_6D_6, 298 K).

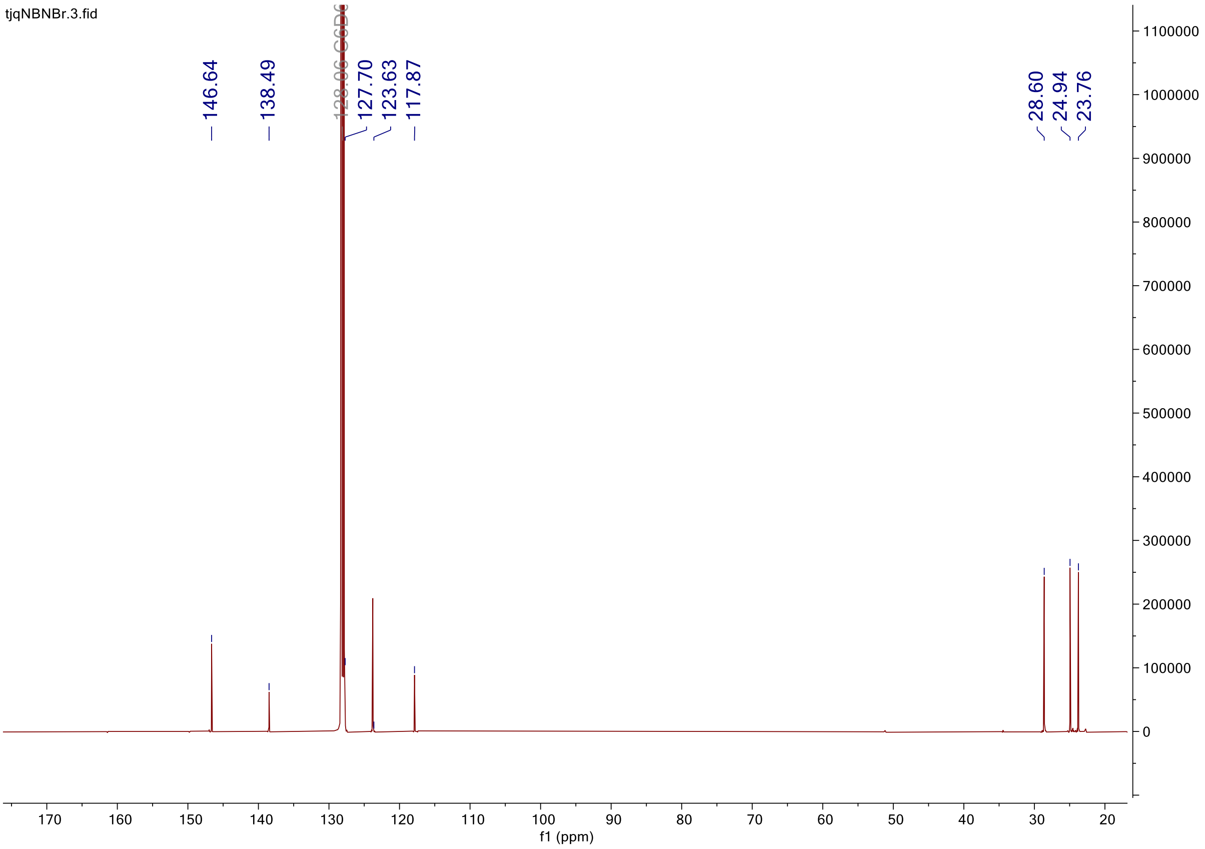

Figure S2. ^{13}C NMR spectrum of **1** (126 MHz, C_6D_6, 298 K).

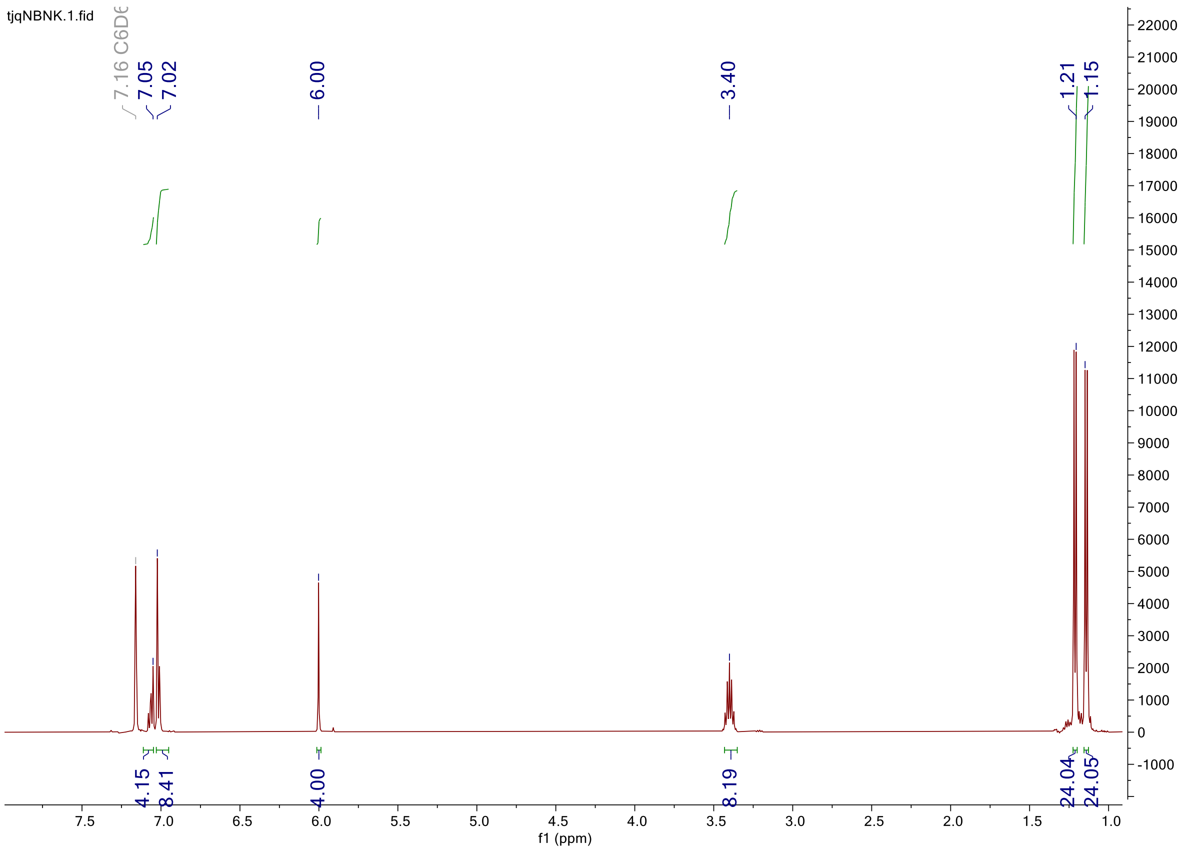

Figure S3. ^{1}H NMR spectrum of **2** (500 MHz, C_6D_6, 298 K).

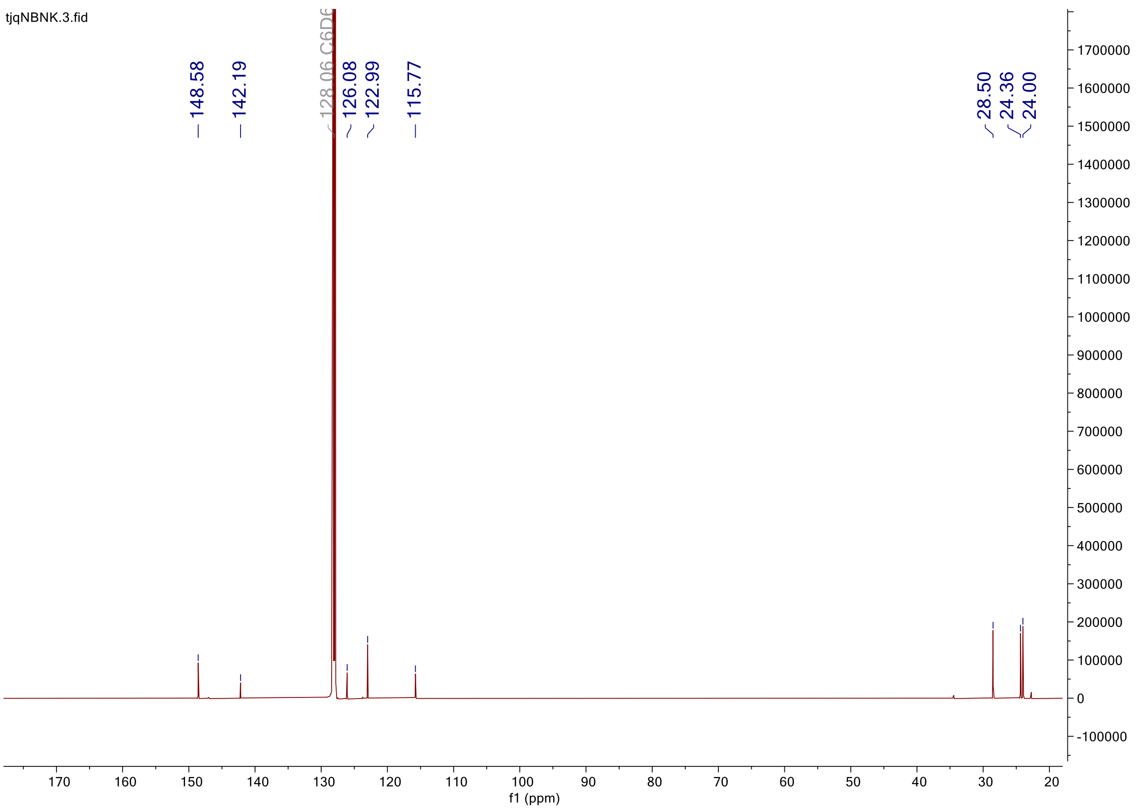

Figure S4. $^{13}C\{^{1}H\}$ NMR spectrum of **2** (126 MHz, C_6D_6, 298 K).

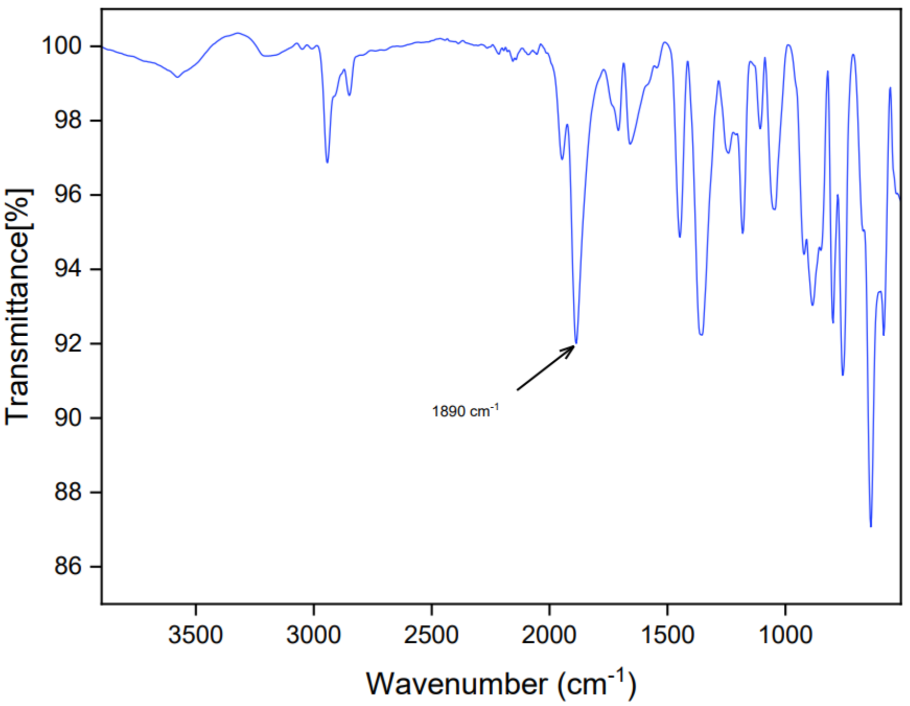

Figure S5. IR spectrum of **3**.

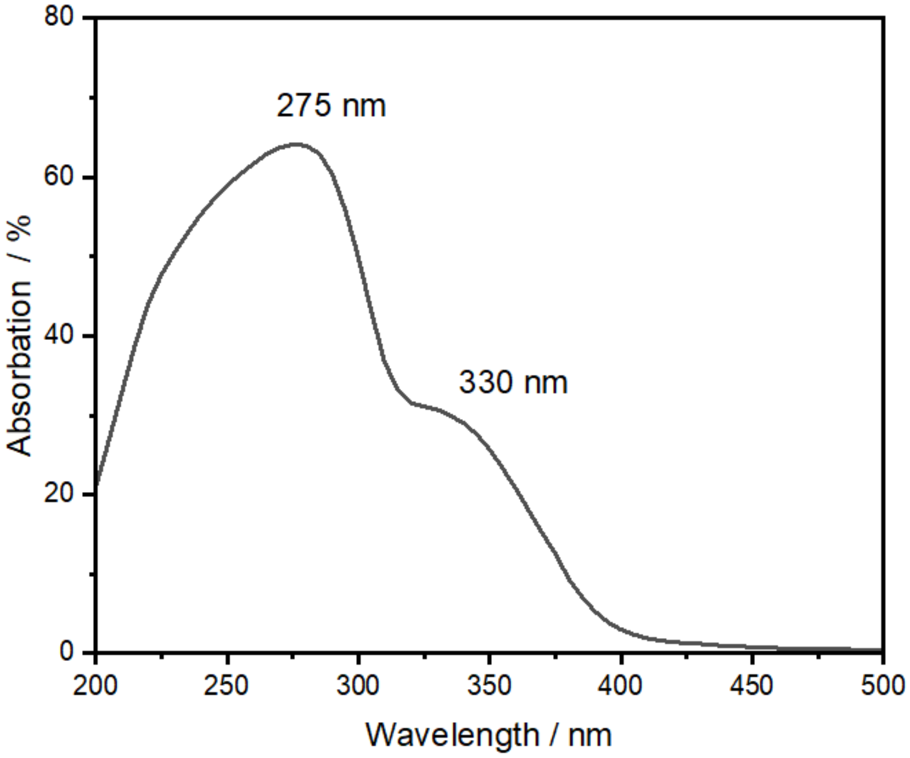

Figure S6. UV-vis spectrum of **3** in hexane.

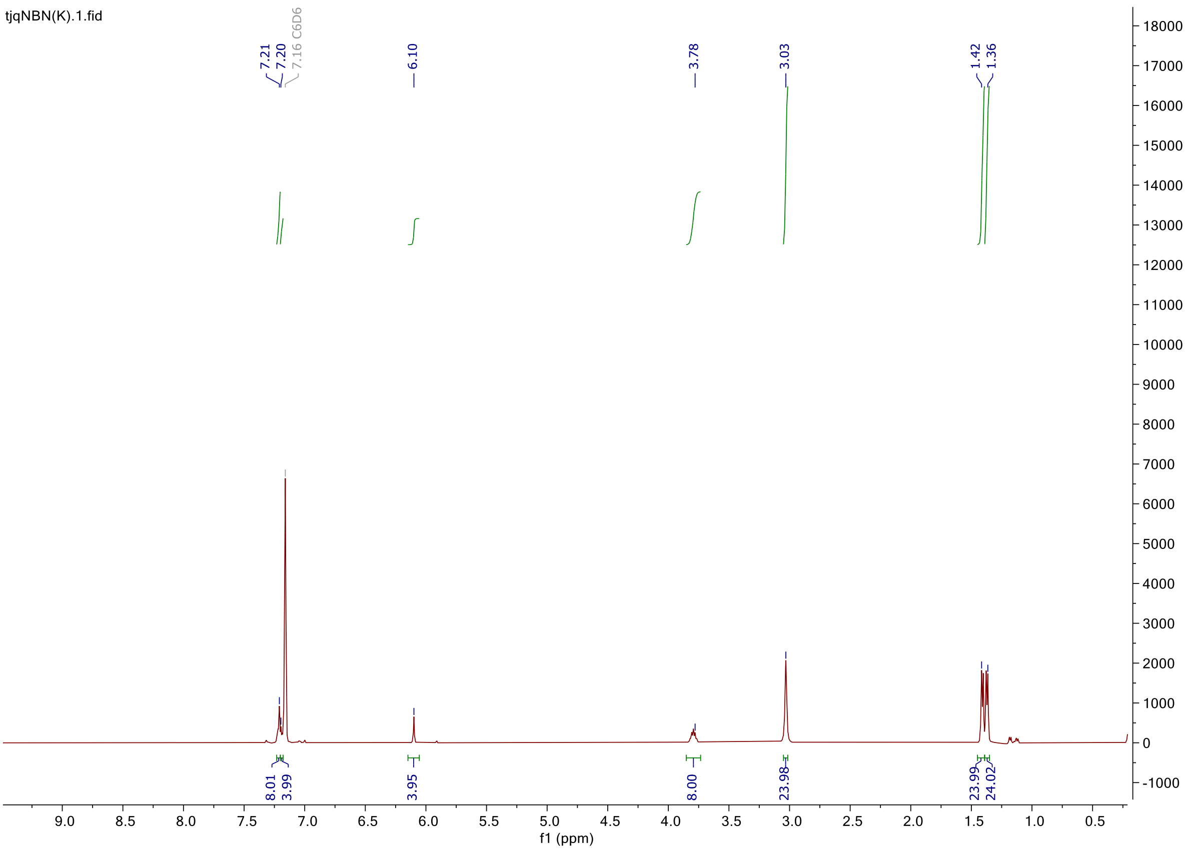

Figure S7. ^{1}H NMR spectrum of **3** (500 MHz, C_6D_6, 298 K).

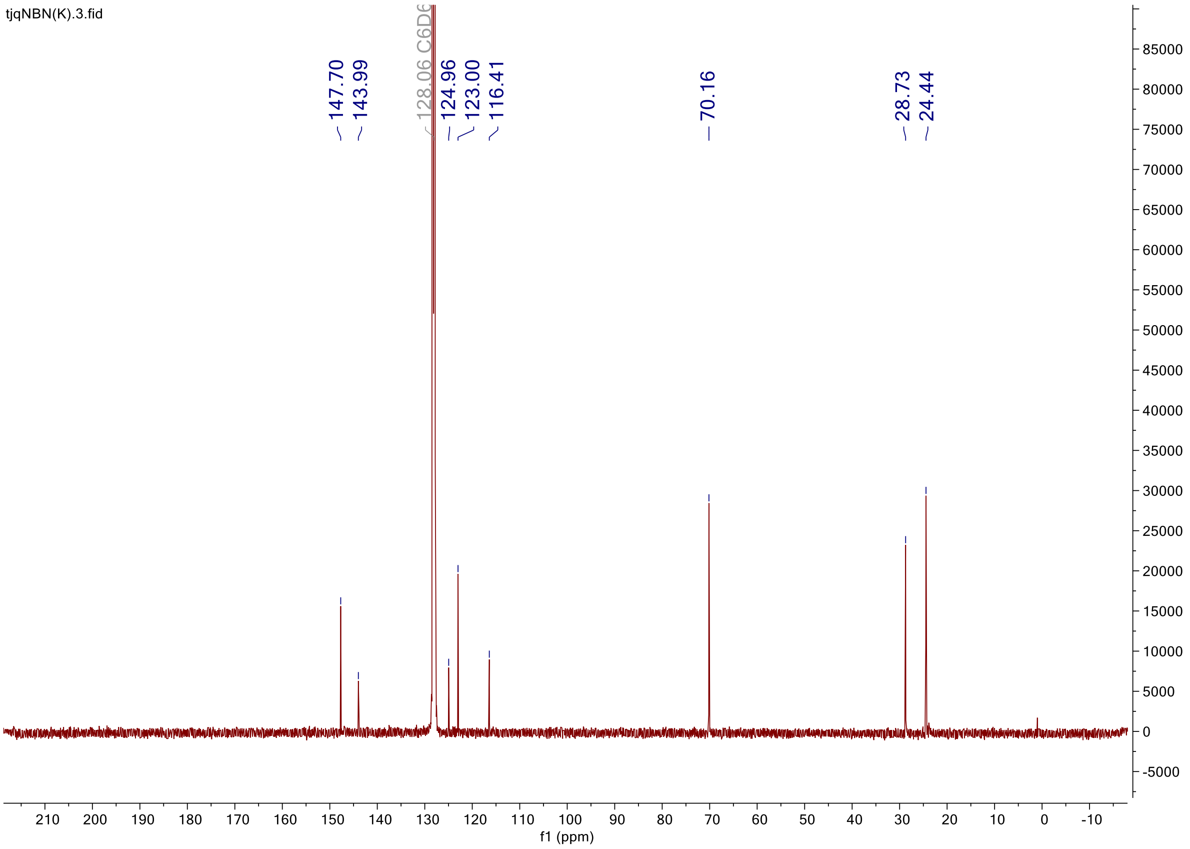

Figure S8. $^{13}C\{^1H\}$ NMR spectrum of **3** (126 MHz, C_6D_6, 298 K).

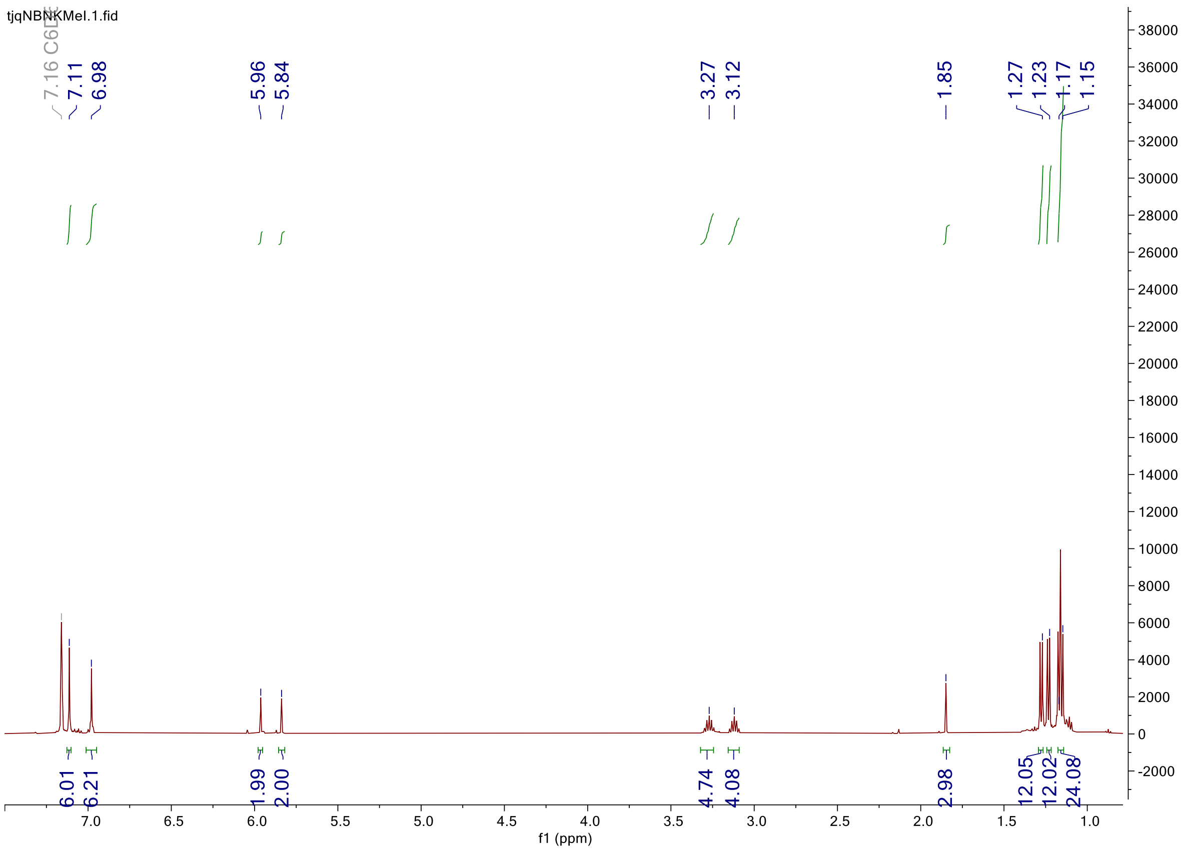

Figure S9. ^{1}H NMR spectrum of **4** (500 MHz, C_6D_6, 298 K).

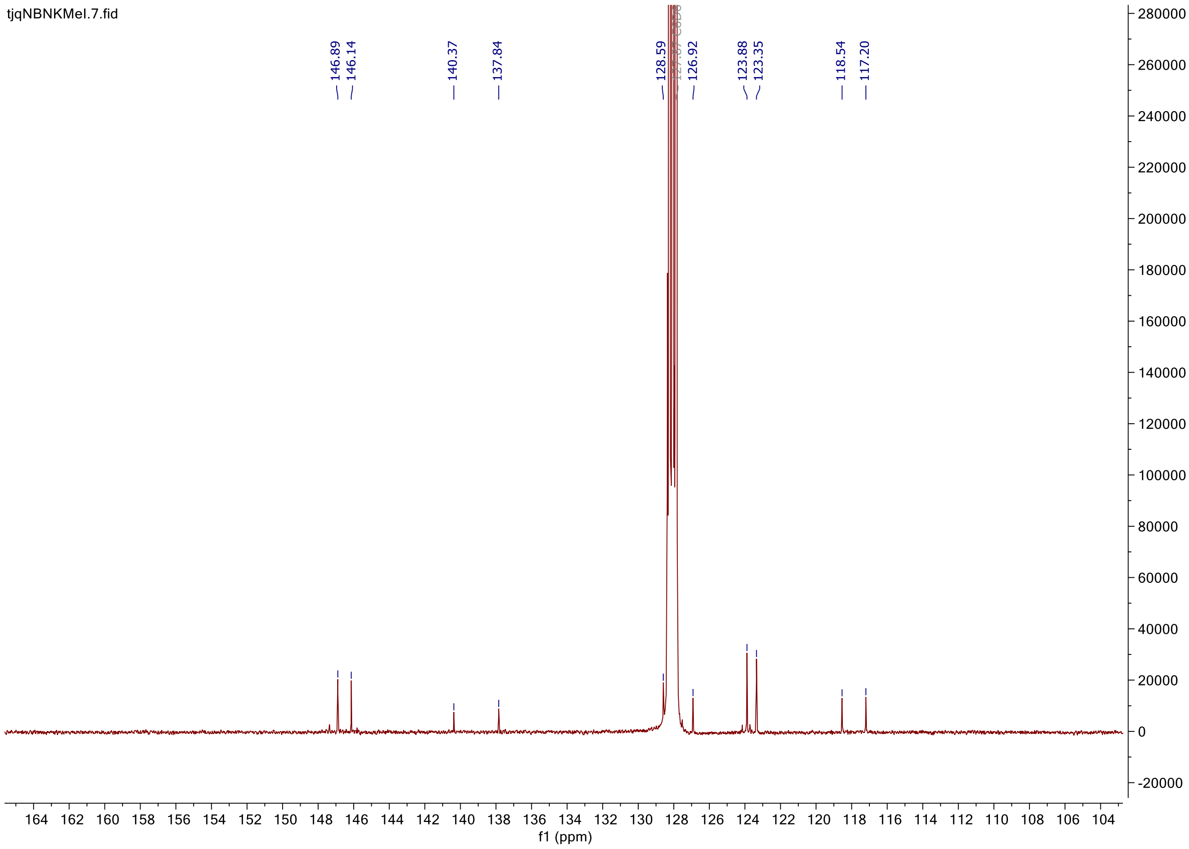

Figure S10. ^{13}C{^{1}H} NMR spectrum of **4** (126 MHz, C_6D_6, 298 K).

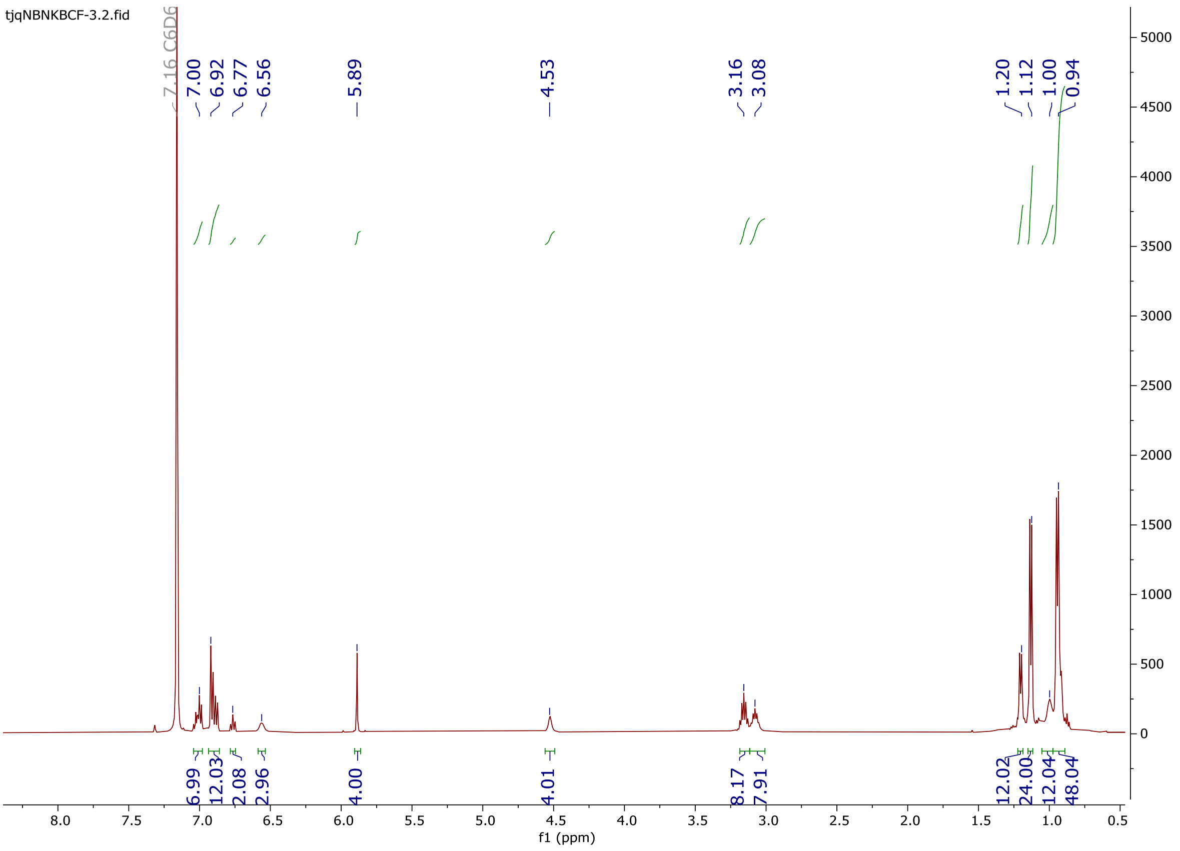

Figure S11. ^{1}H NMR spectrum of **5** (500 MHz, C_6D_6, 298 K).

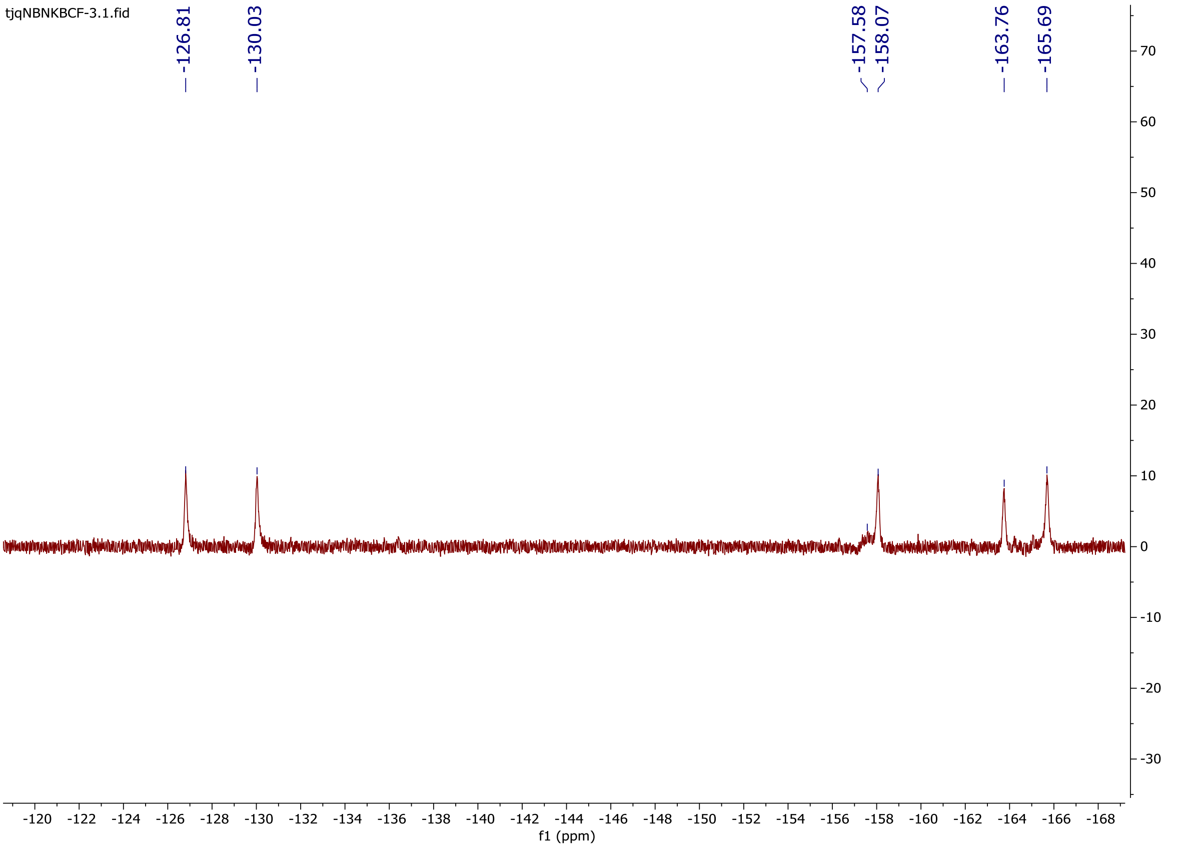

Figure S12. $^{19}F\{^{1}H\}$ NMR spectrum of **5** (470 MHz, C_6D_6, 298 K).

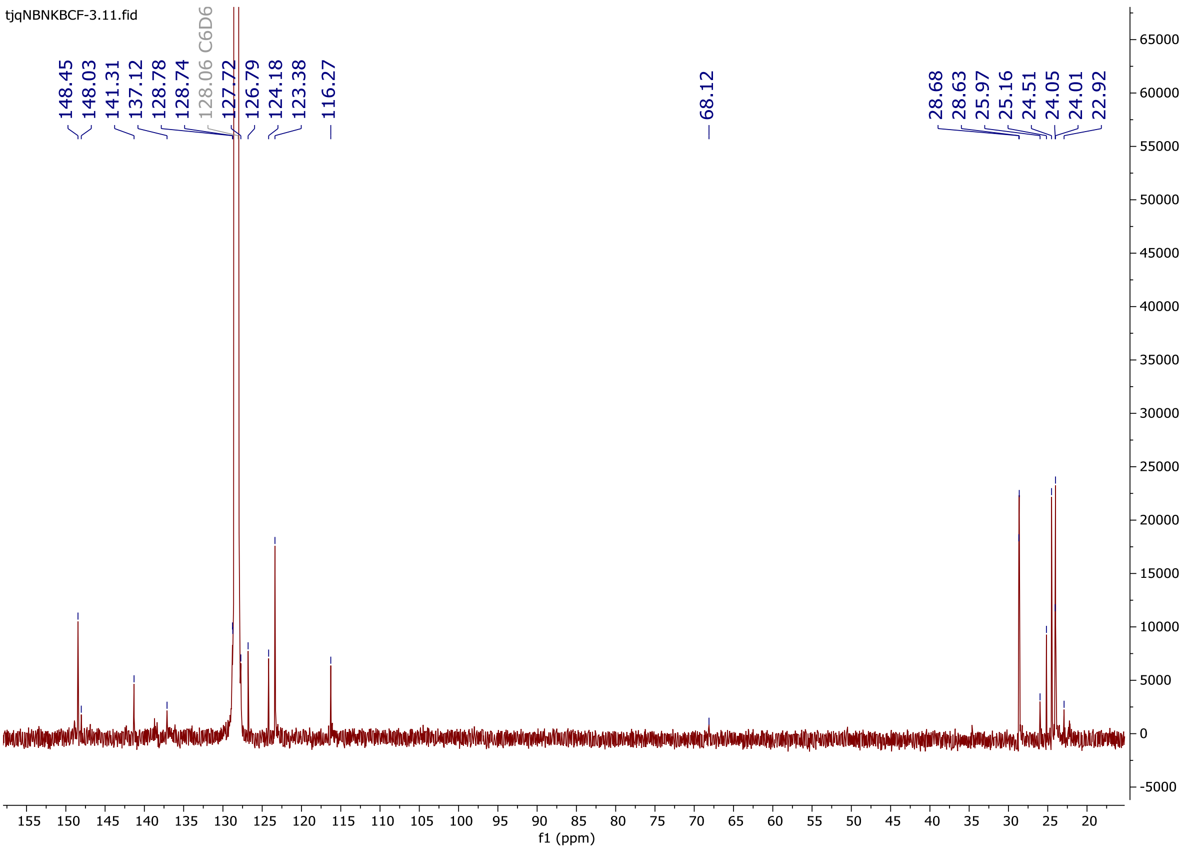

Figure S13. $^{13}C\{^1H\}$ NMR spectrum of **5** (126 MHz, C_6D_6, 298 K).

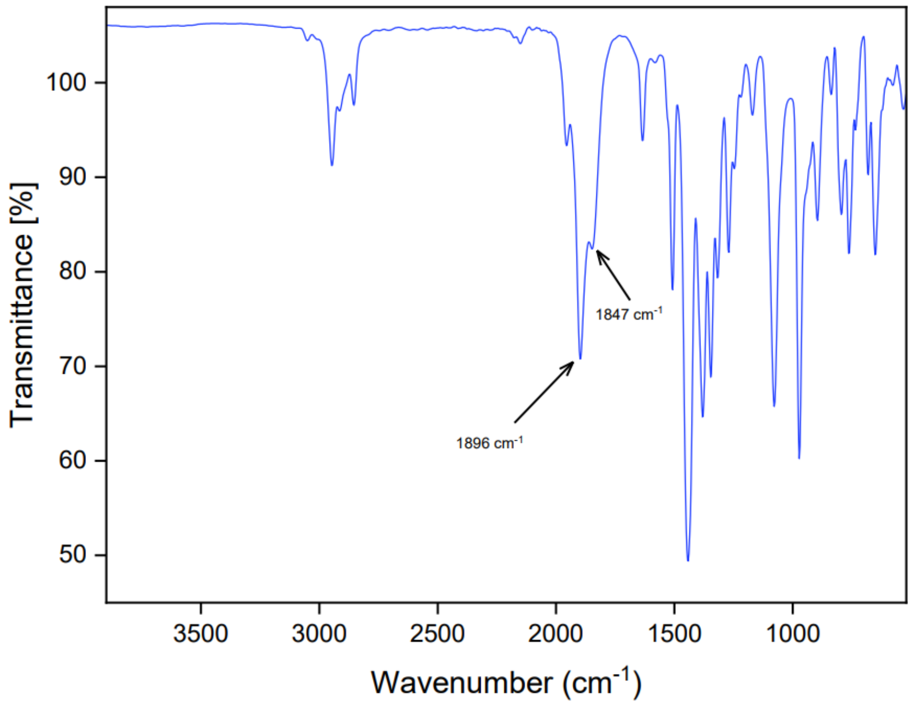

Figure S14. IR spectrum of **5**.

4. Details of X-ray crystallography

Single-crystal X-ray diffraction data for all compounds were collected on an Oxford Diffraction/Agilent SuperNova diffractometer equipped with a 135 mm Atlas CCD area detector or a Rigaku XtaLAB Synergy-DW VHF equipped with a PhotonJet-R dual wavelength rotating anode and HyPix-Arc 150° detector. Crystals were selected under Paratone-N oil, mounted on MiTeGen Micromount loops and quench-cooled using an Oxford Cryosystems open flow N_2 cooling device.[2] Data were collected at 150 K using mirror monochromated Cu Kα radiation (λ = 1.5418 Å). Data collected were processed using the CrysAlisPro package, including unit cell parameter refinement and inter-frame scaling (which was carried out using SCALE3 ABSPACK within CrysAlisPro).[3] Equivalent reflections were merged and diffraction patterns processed with the CrysAlisPro suite.[3] Structures were solved ab initio from the integrated intensities using SHELXT[4] and refined on F2 using SHELXL[5] with the graphical interface OLEX2[6]. Selected crystallographic data are summarised in Table S1-S5, and full details are given in the supplementary deposited CIF files (2377734-37 and 2379472. These data can be obtained free of charge from the Cambridge Crystallographic Data Centre via http://www.ccdc.cam.ac.uk/data_request/cif.

Table S1. Selected crystallographic data and refinement parameters for compounds **1** and **2**.

**Compound**	**1**	**2**
**Empirical formula**	$C_{104}H_{148}B_6Br_2N_{12}$	$C_{55}H_{75}B_3KN_6$
**Fw /g mol^{-1}**	1791.02	891.74
**T/K**	150.15	100.00(10)
**Cell setting**	triclinic	monoclinic
**Space group**	P-1	$P2_1/c$
**a/Å**	12.7280(5)	10.91294(5)
**b/Å**	19.2851(10)	17.73369(8)
**c/Å**	22.9944(10)	27.28930(12)
**α/°**	109.798(4)	90
**β/°**	96.222(3)	93.2201(4)
**γ/°**	93.315(4)	90
**V/Å^3**	5252.3(4)	5272.87(4)
**Z**	2	4
**ρ_{calc}g/cm^3**	1.132	1.123
**μ/mm^{-1}**	1.347	1.18
**Radiation**	CuKα (λ = 1.54184)	Cu Kα (λ = 1.54184)
**Reflections collected**	83631	216448
**Independent reflections**	21692 [R_{int} = 0.1084]	10932 [R_{int} = 0.0681]
**parameters**	1149	650
**GooF**	1.019	1.047
**Final R indexes [I>=2σ (I)]**	R_1 = 0.0725, wR_2 = 0.1844	R_1 = 0.0394, wR_2 = 0.1045
**Final R indexes [all data]**	R_1 = 0.1344, wR_2 = 0.2279	R_1 = 0.0404, wR_2 = 0.1053
**CCDC ref**	2377734	2377735

Table S2. Selected crystallographic data and refinement parameters for compounds **3** and **4**.

Compound	3	4
**Empirical formula**	$C_{64}H_{96}B_3KN_6O_6$	$C_{53}H_{75}B_3N_6$
**Fw /g mol^{-1}**	1116.99	828.62
**T/K**	150.15	100.00(10)
**Cell setting**	monoclinic	monoclinic
**Space group**	$P2_1$	Pn
**a/Å**	12.94630(10)	10.68070(10)
**b/Å**	19.3382(2)	19.8291(2)
**c/Å**	13.9330(2)	11.88910(10)
**α/°**	90	90
**β/°**	107.7870(10)	94.6990(10)
**γ/°**	90	90
**V/Å^3**	3321.50(7)	2509.51(4)
**Z**	2	2
**ρ_{calc}g/cm^3**	1.117	1.097
**μ/mm^{-1}**	1.096	0.476
**Radiation**	CuKα (λ = 1.54184)	Cu Kα (λ = 1.54184)
**Reflections collected**	41193	59412
**Independent reflections**	11552 [R_{int} = 0.0388]	8976 [R_{int} = 0.0363,]
**parameters**	737	607
**GooF**	1.033	1.087
**Final R indexes [I>=2σ (I)]**	R_1 = 0.0411, wR_2 = 0.1046	R_1 = 0.0392, wR_2 = 0.1027
**Final R indexes [all data]**	R_1 = 0.0458, wR_2 = 0.1092	R_1 = 0.0431, wR_2 = 0.1049
**CCDC ref**	2377736	2377737

Table S3. Selected crystallographic data and refinement parameters for compound **5**.

**Compound**	**5**
**Empirical formula**	$C_{140}H_{144}B_8F_{30}K_2N_{12}$
**Fw /g mol^{-1}**	2729.34
**T/K**	150(2)
**Cell setting**	triclinic
**Space group**	P-1
**a/Å**	12.0813(3)
**b/Å**	24.6931(7)
**c/Å**	27.2427(8)
**α/°**	106.582(2)
**β/°**	96.906(2)
**γ/°**	98.386(2)
**V/Å^3**	7592.8(4)
**Z**	2
**ρ_{calc}g/cm^3**	1.194
**μ/mm^{-1}**	1.286
**Radiation**	Cu Kα (λ = 1.54184)
**Reflections collected**	108216
**Independent reflections**	29816 [R_{int} = 0.0229]
**parameters**	1865
**GooF**	0.933
**Final R indexes [I>=2σ (I)]**	R_1 = 0.0614, wR_2 = 0.1368
**Final R indexes [all data]**	R_1 = 0.1361, wR_2 = 0.1750
**CCDC ref**	2379472

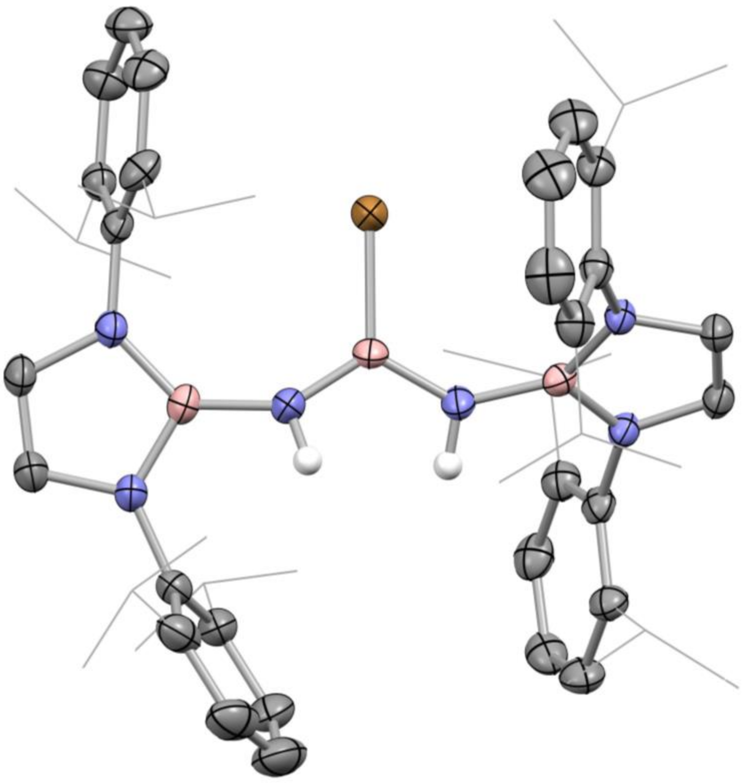

Figure S15. Molecular structure of one of the two independent molecules in the asymmetric unit of **1** in the solid state as determined by X-ray crystallography. Most Hs omitted and ^{i}Pr groups shown in wireframe format for clarity; thermal ellipsoids shown at the 35% probability level.

5. Details of quantum chemical calculations

Geometry optimizations were carried out using the Gaussian 16 package with the M06-2X functional.[7,8] The def2-SVP basis set was used for all atoms.[9,10] Frequency calculations at same level of theory were performed to identify the number of imaginary frequencies (zero for local minimum and one for transition states), and provide the thermal corrections of Gibbs free energy. Single-point energy calculations were performed at the M06-2X/def2-TZVP level of theory for the modelling of compounds in solution (benzene).[11,12] The gas-phase geometry was used for all the solution phase calculations. The SMD method was used with the corresponding solvent, while Bondi radii were chosen as the atomic radii to define the molecular cavity.[13,14] The corrections of Gibbs free energy from frequency calculations were added to the single-point energies to obtain the Gibbs free energy in solution. All energies reported in the paper correspond to the reference state of 1 mol/L, 298 K. Natural bond orbital (NBO) calculations were carried out using NBO 7.0 program at the M06-2X/def2-TZVP level of theory.[15] The Mayer bond order calculation was performed using Multiwfn 3.8.[16] Optimized structures and orbitals were visualized using ChimeraX.[17] The bent isomer of $(NH_2)_2BNBNB(NH_2)_2$ was optimized by freezing the coordinates for all N and B atoms from the optimized geometry of **3**. The energy scans for the BNB bond angles in compounds **3** and **5** (Figure S16) were performed using the optimized geometry of **3**/**5** as a starting point, and performing constrained geometry optimizations at 5º intervals from 115º to 175º. Single-point energy calculations were performed at the M06-2X/def2-TZVP level of theory using these geometries in solution (benzene) to obtain E.

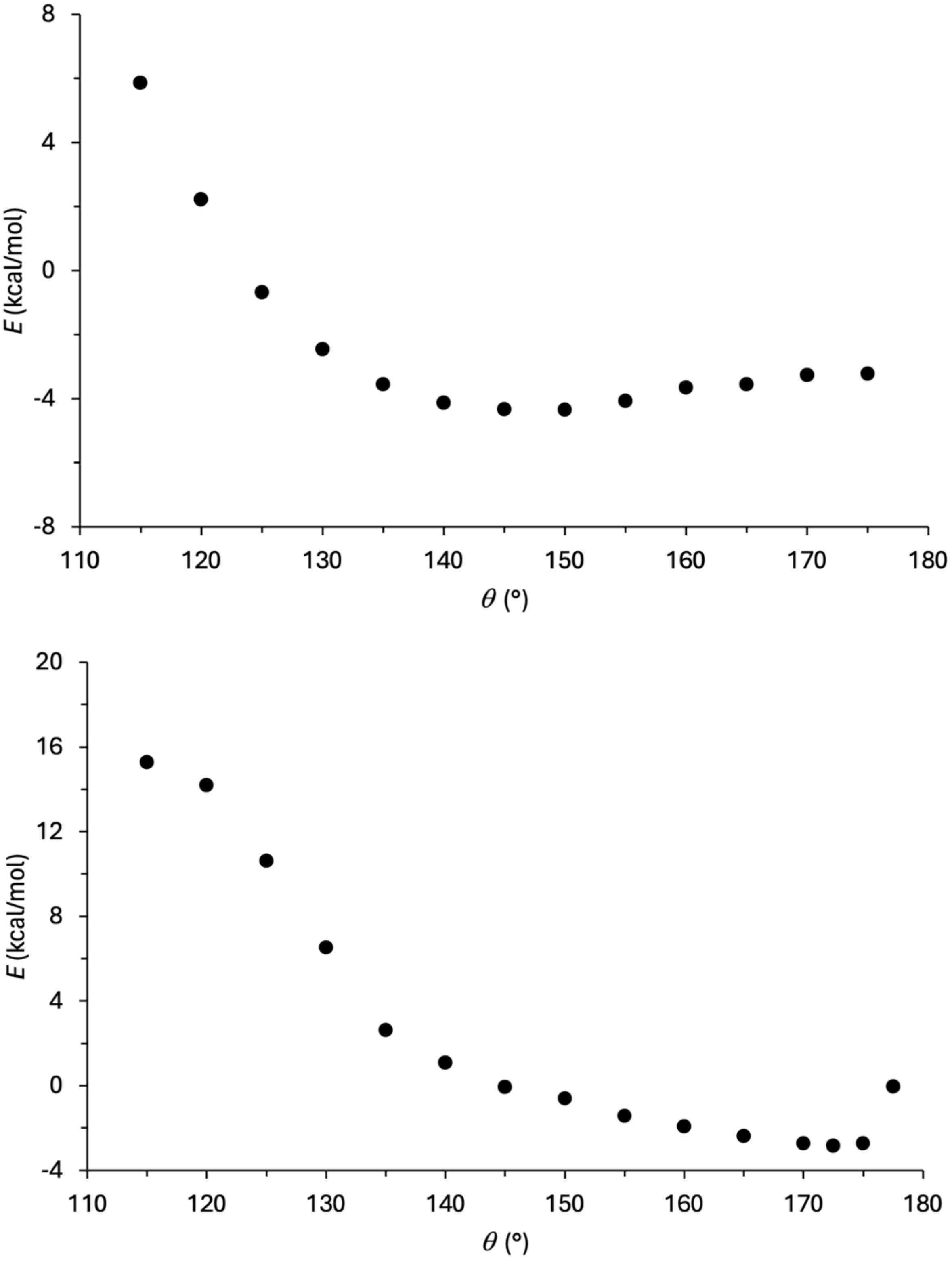

Figure S16. Energy scans for the BNB bond angles (θ) for the anionic components of compounds **3** (upper) and **5** (lower).

Coordinates for optimised geometries

$(NH_2)_2BNBNB(NH_2)_2$ bent isomer

N	-3.67276900	0.90021500	0.51136400
N	3.65015500	-0.91838000	0.51210400
N	-3.23409600	-1.19620100	-0.30669100
N	-1.24439700	0.44051300	-0.18592700
N	1.24170000	-0.41711200	-0.19899700
N	3.26079300	1.18892100	-0.30018400
B	-0.00235000	0.00849400	-0.23036800
B	2.56836600	-0.07826600	-0.01893200
B	-2.57043300	0.08599100	-0.02165200
H	3.46972500	-1.91368700	0.59161900
H	4.56116100	-0.75549100	0.10156600
H	3.99527300	1.41949300	0.35824300
H	2.67992300	2.00269400	-0.47481900
H	-3.51467500	1.89897300	0.59679300
H	-4.57674000	0.72100800	0.09189300
H	-3.96795900	-1.44314300	0.34639700
H	-2.63432400	-1.99664500	-0.47861800

$(NH_2)_2BNBNB(NH_2)_2$ linear isomer

N	-1.31090100	-0.00090900	-0.00094700
N	1.31087900	-0.00044600	-0.00057000
B	-0.00000700	-0.00063300	-0.00070200
B	-2.69570100	-0.00015300	-0.00009400
B	2.69570800	0.00009400	0.00009500
N	-3.49086100	0.92169200	-0.82344700
H	-2.97343700	1.35460300	-1.57897400
H	-4.40007200	0.60326200	-1.14015200
N	-3.49095700	-0.92108700	0.82411700
H	-2.97338700	-1.35473800	1.57910500
H	-4.39965000	-0.60176800	1.14137900
N	3.49018500	0.82497600	0.92115800
H	4.39924400	1.14183800	0.60246100
H	2.97226500	1.58052300	1.35341300
N	3.49162000	-0.82418200	-0.92028600
H	4.40062500	-1.14046700	-0.60084700
H	2.97465000	-1.58009400	-1.35306300

3

N	1.06648810	1.77638766	7.63183186
N	1.80602670	2.95921533	5.82060715
N	3.80788969	6.89247790	11.23329598
N	3.98700756	5.28874733	12.84726177
N	2.47824240	3.90634216	8.09399258
N	3.24241866	4.45760594	10.54901793
C	0.63934024	1.34842645	8.91559920
C	-0.69885124	1.56216740	9.30311570
C	3.91622280	4.08457864	13.59064562
C	2.37931650	3.92120314	4.95156989
C	3.55787884	7.62722021	10.04663429
C	0.60841265	1.16800450	6.45476001
H	0.00701365	0.26834598	6.47477509
C	4.48761072	7.58361931	8.99295247
C	1.05267209	1.86569972	5.38056280
H	0.90120717	1.65828956	4.32938210
C	-1.12114279	1.06991219	10.53981570
H	-2.14962531	1.21743418	10.85679274
C	4.20659470	7.48716159	12.43652749
H	4.39591940	8.54981907	12.51488591
C	5.09437376	3.35219704	13.83466216
C	3.61375139	3.64343020	4.33111351
C	1.56185006	0.74038298	9.78929350
C	1.73259621	5.15601007	4.76855476
C	-1.61468710	2.42214579	8.45048601
H	-1.32177950	2.29715373	7.40190810
C	-0.23357203	0.41888695	11.38726274
H	-0.57481404	0.04781589	12.35038513
C	4.31158819	6.53409095	13.39704471
H	4.60274919	6.64651143	14.43309844
C	2.12605179	9.07382825	8.75343208
H	1.20535240	9.63840053	8.63691658
C	2.66175330	3.61065756	14.01284742
C	2.35543823	8.35509418	9.92666112
C	1.10011705	0.27955392	11.02420737
H	1.79210787	-0.18964758	11.71685357
C	4.21088492	8.30746318	7.82919332
H	4.91677048	8.28074750	7.00272510

C	0.40592264	5.46544125	5.43506598	H	2.88231130	-1.57446473	8.99274397
H	0.07822452	4.55879368	5.95531667	C	0.72230870	4.80593821	15.08297558
C	3.56335102	5.85612035	3.34204492	H	0.39206526	3.92728790	15.64997097
H	4.02990698	6.61422568	2.71801300	H	-0.15798772	5.42925491	14.88649200
C	4.18845721	4.62607911	3.52412689	H	1.41407622	5.37594680	15.71303858
H	5.14307744	4.43809354	3.04111582	C	0.41524506	3.61354940	12.87254319
C	3.04857853	9.05516032	7.71236060	H	0.89530028	3.32037905	11.93378588
H	2.84871590	9.61098757	6.79980189	H	-0.46226262	4.22664273	12.63434502
C	1.38860369	4.39603483	13.76202371	H	0.06476253	2.70326837	13.37234614
H	1.66284820	5.31261326	13.22874906	C	0.56357179	6.57430401	6.48343069
C	2.34804013	6.11621986	3.96004360	H	0.88002073	7.51527926	6.01777172
H	1.86926988	7.08206648	3.81677348	H	1.31179241	6.29372672	7.23090571
C	4.33198879	2.33649404	4.61449777	H	-0.38923557	6.75306683	6.99654853
H	3.56810211	1.57806007	4.82277155	C	-0.67273289	5.82763542	4.40513820
C	4.99416929	2.13548123	14.51076173	H	-1.63854133	5.96735038	4.90473525
H	5.88802937	1.54851291	14.70238467	H	-0.78535327	5.03711346	3.65487164
C	5.77657779	6.79163232	9.09593723	H	-0.43088292	6.76005327	3.88142620
H	5.75285231	6.24290705	10.04320233	B	2.83188598	4.19119868	9.32939322
C	2.60469037	2.38292349	14.67886018	C	3.97773211	0.48163504	10.55882671
H	1.64228942	1.99529328	15.00497631	H	3.85819933	-0.45270912	11.12062866
C	6.42190325	3.84207036	13.28675579	H	3.83870597	1.32262332	11.24743338
H	6.36519999	4.93424349	13.21374933	H	5.00920036	0.51331186	10.19096083
C	3.75792738	1.65108191	14.92739857	C	0.45286980	9.53528586	11.15455912
H	3.69563065	0.69669164	15.44422059	H	1.08355491	10.42244637	11.28060488
C	1.29438567	8.26441148	11.00904770	H	-0.19771099	9.44923050	12.03185292
H	1.80216308	8.09192008	11.96505325	H	-0.19436076	9.69744445	10.28519671
C	3.01165272	0.56597559	9.37491696	B	3.63597993	5.44811796	11.43390325
H	3.28846355	1.45358865	8.79391182	C	5.89943933	5.75985525	7.96708828
C	-3.09611474	2.05387612	8.56302438	H	5.02135803	5.10710335	7.93933715
H	-3.50008858	2.28783170	9.55453457	H	6.78981631	5.13829004	8.12029847
H	-3.26053920	0.98793649	8.36972728	H	5.99382843	6.24382546	6.98717950
H	-3.67624828	2.62837946	7.83247767	B	1.84813465	2.97382698	7.28599872
C	6.99301300	7.72816285	9.12617891	C	0.39289660	7.04664958	10.73927934
H	7.07600146	8.29553054	8.19128163	H	-0.13594477	7.16976646	9.78575868
H	7.91617456	7.15063428	9.25416920	H	-0.35276238	6.93536027	11.53627419
H	6.91827565	8.44489452	9.95165853	H	0.98011018	6.12398647	10.68282975
C	3.16278580	-0.65832889	8.45799840	C	7.61768290	3.49521164	14.17783127
H	4.20231580	-0.75984122	8.12391485	H	7.81672534	2.41766974	14.19212322
H	2.52536033	-0.56513468	7.57295902	H	7.45859696	3.82623588	15.21018586

H	8.51911266	3.98637615	13.79495600
C	6.62753475	3.29535633	11.86382616
H	7.54902699	3.69808599	11.42587450
H	5.78558592	3.55901008	11.21469526
H	6.70688153	2.20138516	11.88771340
C	5.17923551	1.83533186	3.44173750
H	5.55772333	0.83118037	3.66249226
H	4.59615123	1.78653566	2.51528391
H	6.04906940	2.47799730	3.26459616
C	5.19207597	2.47968872	5.88148202
H	4.58680380	2.80764260	6.73260561
H	5.66497191	1.52291973	6.13454889
H	5.98308926	3.22328284	5.72177133
C	-1.39418349	3.90080820	8.81827768
H	-0.34042974	4.18253823	8.71509592
H	-1.99294532	4.55282044	8.17016378
H	-1.69268368	4.07979516	9.85903684

5

N	-2.26578991	10.25479708	19.028570271
B	-2.02056023	11.18446975	18.150959205
N	-1.78230566	12.08717936	17.243392445
B	-2.64823571	9.18344807	19.762742897
N	-3.94128358	9.08883459	20.619815481
C	-3.96888004	7.99842659	21.349424187
C	-2.69774325	7.23081082	21.075512484
H	-2.08712415	7.19074047	21.990760777
H	-2.93882850	6.18274853	20.836668257
N	-2.01349210	7.93759776	20.020591630
B	-5.04148589	7.30681841	22.410413212
C	-4.91878593	10.14876391	20.479294123
C	-5.00155446	11.15164844	21.460230174
C	-5.91898879	12.18397111	21.257882005
H	-6.01889780	12.96255257	22.007262027
C	-6.69741994	12.23870583	20.110241505
H	-7.40938456	13.04835442	19.974991085
C	-6.54196017	11.27722841	19.124350199
H	-7.12785752	11.34622861	18.212161897
C	-5.63720841	10.22527146	19.272915405
C	-4.07015179	11.17664975	22.654881541
H	-3.84372096	10.14363258	22.920301537
C	-4.67579649	11.84109341	23.893746399
H	-4.73732451	12.92922087	23.779632529
H	-4.04307457	11.63896279	24.764699884
H	-5.67956756	11.46148810	24.104327236
C	-2.74986823	11.87337182	22.282369170
H	-2.27480786	11.41801240	21.408315265
H	-2.04915076	11.83174016	23.124684906
H	-2.93680525	12.92747428	22.043292257
C	-5.49277027	9.22902688	18.133540717
H	-4.69526590	8.52275005	18.380997744
C	-5.09549705	9.93322857	16.826701273
H	-4.89852830	9.19013025	16.045551887
H	-4.19408766	10.53862930	16.956196334
H	-5.89353233	10.59105576	16.465024734
C	-6.78352447	8.42288664	17.920079911
H	-7.59995730	9.08000997	17.599105922
H	-7.10431888	7.91433434	18.832235800
H	-6.62955106	7.66871268	17.139961815
C	-0.68022740	7.58345418	19.664378086
C	-0.47296124	6.76971014	18.534467696
C	0.83817388	6.44668075	18.182962215
H	1.02596936	5.82871798	17.309906237
C	1.91282490	6.91240234	18.932408252
H	2.92776182	6.65111587	18.644276847
C	1.69257734	7.72145409	20.038229384
H	2.54023746	8.09236170	20.609172605
C	0.39675370	8.07777572	20.421581611
C	-1.64661952	6.32816330	17.681080019
H	-2.52836938	6.28207970	18.330298848
C	-1.45894643	4.94528299	17.051128758
H	-2.38937248	4.63217713	16.565303339
H	-1.19473975	4.19382289	17.803153199
H	-0.67691427	4.95079586	16.283810843
C	-1.92718116	7.38369182	16.597808393
H	-1.07155996	7.46231317	15.915781964
H	-2.09626189	8.37098915	17.039535697
H	-2.81081255	7.10904022	16.009575115

C	0.19479172	9.00146723	21.608641293
H	-0.88034274	9.16785000	21.739218731
C	0.83197380	10.37336254	21.347543886
H	1.91973184	10.29195906	21.240165493
H	0.62484309	11.05373587	22.181569501
H	0.43422310	10.82188126	20.431320750
C	0.72792187	8.37969001	22.906523327
H	0.26653319	7.40408968	23.098821600
H	0.50922459	9.03258069	23.758912973
H	1.81343958	8.23506866	22.859782371
B	-1.39815065	13.01154831	16.332957718
N	-0.14076820	13.90882521	16.437453821
C	-0.07233585	14.78492363	15.464190317
C	-1.29611756	14.60183626	14.597411478
H	-1.92191794	15.50616484	14.664743293
H	-1.00298764	14.52426323	13.538523932
N	-1.98507652	13.44226219	15.110512069
B	0.99667769	15.96618810	15.007997835
C	0.77316228	13.68402364	17.536145586
C	0.64112408	14.45620441	18.701276796
C	1.55500071	14.23122771	19.733009249
H	1.49241816	14.82849791	20.638103413
C	2.51920657	13.23840952	19.631601590
H	3.22870993	13.08486451	20.440262135
C	2.53808059	12.40373078	18.523266842
H	3.24787708	11.58205416	18.483659008
C	1.64821524	12.58579274	17.464052620
C	1.62738325	11.56648159	16.334796840
H	0.85290606	11.85221917	15.616480253
C	1.25393820	10.18006592	16.888097925
H	1.12674341	9.47162346	16.061475536
H	0.32219118	10.21004913	17.459327913
H	2.04020931	9.79027677	17.544329175
C	2.95743392	11.47032921	15.573907368
H	2.88003778	10.70253458	14.795916032
H	3.77329610	11.18430910	16.247453779
H	3.23055291	12.41257988	15.095749497
C	-0.52917354	15.39445189	18.931163171
H	-1.04656462	15.54647853	17.981845461

C	-0.11909714	16.76872569	19.468053674
H	-0.99646167	17.42180215	19.526733838
H	0.30569055	16.69589757	20.475355603
H	0.61885837	17.24695137	18.819198218
C	-1.52758136	14.72348676	19.892718503
H	-2.40529007	15.36574679	20.031901302
H	-1.86294655	13.75739251	19.502996665
H	-1.06723737	14.55565088	20.873579425
C	-3.28921207	13.10405180	14.650656993
C	-3.42648550	12.09704879	13.677160803
C	-4.71274744	11.74854052	13.262763410
H	-4.84732366	10.96850185	12.519194061
C	-5.82989449	12.38222269	13.796341661
H	-6.82460069	12.09808634	13.462848579
C	-5.67787043	13.36685268	14.762753642
H	-6.55854800	13.84512032	15.184430941
C	-4.40902273	13.74138164	15.212934881
C	-2.20500614	11.36313797	13.156992182
H	-1.35103394	12.04400136	13.247298534
C	-2.31809127	10.94995716	11.687260878
H	-2.58931523	11.79972374	11.051400952
H	-3.06399498	10.16073123	11.540819570
H	-1.35606576	10.55653980	11.341377964
C	-1.91019449	10.14199338	14.044507861
H	-2.73977777	9.42554333	13.996552060
H	-1.77783841	10.43384267	15.091482597
H	-0.99906048	9.63422704	13.706362296
C	-4.27722674	14.78952017	16.302278716
H	-3.21270849	14.92179873	16.525686525
C	-4.95056239	14.32054712	17.599040965
H	-4.81107742	15.06365521	18.392703758
H	-6.02788880	14.17585199	17.458743517
H	-4.52527149	13.37051902	17.939133052
C	-4.82634552	16.14869046	15.846298167
H	-4.65301188	16.90747208	16.617410544
H	-4.33877033	16.48164620	14.922646638
H	-5.90481519	16.09689163	15.657559589
C	0.00904526	17.14889039	14.381004106
C	-0.97066681	17.71131447	15.200643883

C	-1.88328231	18.67225381	14.803857258
C	-1.86524699	19.11257884	13.487580661
C	-0.93618206	18.57308944	12.616622243
C	-0.03655254	17.60800887	13.066555450
F	-1.06788546	17.29740525	16.482990401
F	-2.78231357	19.17181279	15.663794217
F	-2.73690042	20.03522696	13.067321046
F	-0.91663015	18.97469494	11.337176213
F	0.78570717	17.12710592	12.112029549
C	2.00502788	15.38107656	13.837579865
C	1.98920267	14.11235746	13.275360100
C	2.91790626	13.66440734	12.342371583
C	3.93129077	14.51196091	11.929358498
C	3.99005491	15.79637384	12.453335324
C	3.03993527	16.19669229	13.378583796
F	1.04651022	13.20716068	13.628980529
F	2.84698426	12.41823361	11.851496689
F	4.83611530	14.10279054	11.032643123
F	4.95531755	16.63432654	12.048235458
F	3.12773002	17.47247819	13.808734612
C	1.99173357	16.52273725	16.210509772
C	2.01822108	17.80489296	16.761039532
C	2.92652852	18.21508097	17.731475648
C	3.89824438	17.34010564	18.184301227
C	3.95890654	16.07186966	17.631612608
C	3.03107258	15.70868048	16.667070659
F	1.16524756	18.77099911	16.362350051
F	2.86920248	19.45919591	18.231313062
F	4.77400481	17.71971173	19.121568804
F	4.92109809	15.22231588	18.017815937
F	3.20870468	14.48632081	16.131444355
C	-6.06807543	8.36208222	23.172733984
C	-7.11828930	8.93479379	22.451193986
C	-8.10611183	9.74178374	22.996280576
C	-8.09922607	9.99661319	24.357268801
C	-7.10445216	9.42780640	25.133669104
C	-6.12716246	8.63612150	24.540504065
F	-7.24930688	8.68109101	21.135120843
F	-9.06624961	10.26797038	22.223071257
F	-9.03344099	10.77791503	24.910466464
F	-7.08249340	9.65910852	26.455235980
F	-5.24070360	8.11026972	25.410522297
C	-4.07214409	6.47179326	23.477397466
C	-3.08618683	7.15667945	24.190955728
C	-2.20529322	6.57873168	25.087500073
C	-2.26675911	5.20950394	25.303881326
C	-3.20671271	4.46854898	24.611521103
C	-4.07057196	5.09841511	23.716916596
F	-2.94145829	8.48914354	24.008047238
F	-1.29434810	7.31659047	25.737511296
F	-1.43003461	4.62050322	26.163597049
F	-3.26834556	3.14129829	24.792499026
F	-4.91371219	4.25905072	23.082252861
C	-6.02122485	6.24905406	21.597951926
C	-7.09328803	5.66508067	22.274917773
C	-8.00845497	4.80820569	21.686025934
C	-7.86967562	4.47949496	20.344332250
C	-6.81208374	5.01426315	19.630152388
C	-5.92354132	5.87556021	20.265071699
F	-7.25655022	5.88776760	23.595808027
F	-9.00982415	4.27270705	22.399099243
F	-8.73788257	3.64799671	19.757381945
F	-6.66278661	4.71505426	18.331419784
F	-4.92477029	6.34746259	19.483161175
F	-4.92477029	6.34746259	19.483161175

6. References for supporting information

[1] T. J. Hadlington, J. A. B. Abdalla, R. Tirfoin, S. Aldridge, C. Jones, *Chem. Commun.* ***52***, 1717–1720 (2016).

[2] J. Cosier, A. M. Glazer, *A Nitrogen-Gas-Stream Cryostat for General X-Ray Diffraction Studies*, (1986).

[3] *CrysAlisPro, Agilent Technologies, Version 1.171.42.57a***.**

[4] G. M. Sheldrick, *Acta Crystallographica Section A: Foundations of Crystallography*, **71**, 3–8 (2015).

[5] G. M. Sheldrick, *Acta Crystallographica Section C: Structural Chemistry,* **71**, 3–8 (2015).

[6] O. V. Dolomanov, L. J. Bourhis, R. J. Gildea, J. A. K. Howard, H. Puschmann, *Journal of Applied Crystallography*, **42**, 339–341 (2009).

[7] Frisch, M. J., Trucks, G. W., Schlegel, H. B., Scuseria, G. E., Robb, M. A., Cheeseman, J. R.; Scalmani, G.; Barone, V.; Petersson, G. A.; Nakatsuji, H.; Li, X.; Caricato, M.; Marenich, A. V.; Bloino, J., Janesko, B. G., Gomperts, R., Mennucci, B., Hratchian, H. P., Ortiz, J. V., Izmaylov, A. F., Sonnenberg, J. L., Williams-Young, D., Ding, F., Lipparini, F., Egidi, F., Goings, J., Peng, B., Petrone, A., Henderson, T., Ranasinghe, D., Zakrzewski, V. G., Gao, J., Rega, N., Zheng, G., Liang, W., Hada, M., Ehara, M., Toyota, K., Fukuda, R., Hasegawa, J., Ishida, M., Nakajima, T., Honda, Y., Kitao, O., Nakai, H., Vreven, T., Throssell, K., Montgomery Jr., J. A., Peralta, J. E., Ogliaro, F., Bearpark, M. J., Heyd, J. J., Brothers, E. N., Kudin, K. N., Staroverov, V. N., Keith, T. A., Kobayashi, R., Normand, J., Raghavachari, K., Rendell, A. P., Burant, J. C., Iyengar, S. S., Tomasi, J., Cossi, M., Millam, J. M., Klene, M., Adamo, C., Cammi, R., Ochterski, J. W., Martin, R. L., Morokuma, K., Farkas, O., Foresman, J. B. & Fox, D. J. Gaussian 16, Revision B.01, Wallingford, CT, (2016).

[8] Zhao, Y & Truhlar, D. G. The M06 Suite of Density Functionals for Main Group Thermochemistry, Thermochemical Kinetics, Noncovalent Interactions, Excited States, and Transition Elements: Two New Functionals and Systematic Testing of Four M06-Class Functionals and 12 Other Functionals. *Theor. Chem. Acc.* **120**, 215−241 (2008).

[9] Weigend, F. & Ahlrichs, R. Balanced Basis Sets of Split Valence, Triple Zeta Valence and Quadruple Zeta Valence Quality for H to Rn: Design and Assessment of Accuracy. *Phys. Chem. Chem. Phys.* **7**, 3297−3305 (2005).

[10] Weigend, F. Accurate Coulomb-Fitting Basis sets for H to Rn. *Phys. Chem. Chem. Phys.* **8**, 1057−1065 (2006).

[11] Schäfer, A.; Horn, H. & Ahlrichs, R. Fully Optimized Contracted Gaussian Basis Sets for Atoms Li to Kr. *J. Chem. Phys.* **97**, 2571−2577 (1992).

[12] Schäfer, A.; Huber, C. & Ahlrichs, R. Fully Optimized Contracted Gaussian Basis Sets of Triple Zeta Valence Quality for Atoms Li to Kr. *J. Chem. Phys.*, **100**, 5829−5835 (1994).

[13] Marenich, A. V.; Cramer, C. J. & Truhlar, D. G. Universal Solvation Model Based on Solute Electron Density and on a Continuum Model of the Solvent Defined by the Bulk Dielectric Constant and Atomic Surface Tensions. *J. Phys. Chem. B*, **113**, 6378−6396 (2009).

[14] Bondi, A. van der Waals Volumes and Radii. *J. Phys. Chem.* **68**, 441–451 (1964).

[15] Glendening, E. D.; Badenhoop, J. K., Reed, A. E., Carpenter, J. E., Bohmann, J. A., Morales, C. M., Karafiloglou P.; Landis C. R. & Weinhold, F., NBO 7.0, University of Wisconsin: Madison, WI (2018).

[16] Lu, T. & Chen, F. Multiwfn: A Multifunctional Wavefunction Analyzer. *J. Comput. Chem*, **33**, 580−592 (2012).

[17] UCSF ChimeraX: Tools for structure building and analysis. Meng EC, Goddard TD, Pettersen EF, Couch GS, Pearson ZJ, Morris JH, Ferrin TE. Protein Sci. 2023 Nov;32(11):e4792.
